# Unveiling Copper‐Induced Phase Transitions and Degradation Mechanisms of Transition Metal Sulfide Anodes for Sodium‐Ion Batteries

**DOI:** 10.1002/advs.202524191

**Published:** 2026-01-28

**Authors:** Jacob Choe, Junpyo Hur, Sanghyeon Park, Jeongmin Kim, Jaeeun Joo, Jae Yeol Park, Chan‐Woo Lee, Jong Min Yuk

**Affiliations:** ^1^ Department of Materials Science & Engineering Korea Advanced Institute of Science and Technology (KAIST) Daejeon Republic of Korea; ^2^ Energy Storage Research Department Korea Institute of Energy Research (KIER) Daejeon Republic of Korea

**Keywords:** conversion reaction, current collector, metal sulfides, phase transition, sodium polysulfide

## Abstract

Transition metal sulfides (TMSs) are promising anodes for sodium‐ion batteries (SIBs) owing to their high theoretical capacity and natural abundance. However, their electrochemical performance is strongly affected by the formation of sodium polysulfides (NaPSs), which induce complex phase transformations and electrode degradation. In particular, NaPSs react with the copper (Cu) current collector, leading to the formation of Cu‐incorporated sulfides, yet the underlying mechanisms remain insufficiently understood. Here, we unveil the fundamental origins of phase evolution in TMS‐based anodes. We reveal that NaPSs formed during early cycling react with Cu substrates to yield NaCu_5_S_3_ and Cu_2_S interfacial phases, initiating a progressive transition toward Cu‐incorporated sulfides. This process is driven by the preferential substitution of Na^+^ in the Na_2_S matrix with Cu ions during desodiation, ultimately evolving into Cu_1.8_S. Furthermore, we demonstrate that the deactivation and ionization of transition metals, followed by their redeposition on the counter electrode, constitute critical degradation pathways. Consequently, our investigation suggests Cu_1.8_S as the ultimate phase of TMS with the most stable phase. Our findings not only establish a mechanistic framework for Cu‐induced phase transitions in TMS anodes but also provide design principles for realizing durable and high‐performance SIB systems.

## Graphical abstract

Sodium polysulfides, which are generated from TMS anodes, react with the Cu current collector, thereby triggering Na‐Cu–S interfacial phase formation. Simultaneously, transition metals undergo ionization and redeposition on the counter electrode. The coupled processes drive the phase evolution of TMS toward the stable Cu_1.8_S phase.

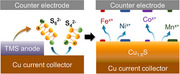

## 1 Introduction

Transition metal sulfides (TMS) have emerged as attractive anode materials for sodium‐ion batteries (SIBs), owing to their abundant availability and intrinsically high theoretical capacities [1, 2, 3]. However, their practical application is hindered by severe volume changes during the conversion‐type sodium storage reactions, leading to mechanical degradation such as pulverization of active material and contact loss with the conductive matrix [4, 5].

While this degradation behavior is critical, another phenomenon has recently attracted growing attention in the field. In recent reports, sodium polysulfides (NaPSs), which are formed through the irreversible and incomplete conversion reaction of Na_2_S, have been observed to react with the copper (Cu) current collector (CC), resulting in the phase conversion toward Cu incorporated sulfide [6, 7, 8, 9, 10, 11, 12]. Several studies have demonstrated that Cu is diffused from the CC forms a Cu*_x_* S phase within the TMS electrode during repeated cycles, thereby contributing to performance enhancement owing to their intrinsic characteristics including high electrical conductivity [7, 13, 14, 15, 16, 17]. However, corrosion and thinning of the CC resulting from this Cu supply during cycling inevitably has an adverse effect in high‐mass loading cases or practical battery systems. Moreover, to fully realize the advantages of SIB systems, such as enhanced energy density and improved stability achieved through the use of aluminum (Al) foil as the anode current collector, it is necessary to explore a system that operates favorably without Cu induced phenomena and complex electrode designs.

Previous studies have primarily explained the principles behind this Cu‐incorporated phase transition using hard soft acid base (HSAB) theory [7, 8, 18, 19]. However, further investigation is required to understand the detailed phase transformation mechanism and how those phase transition affects the electrochemical performance of TMS anodes. Moreover, the failure pathways of existing original transition metal elements, which could affect the stability of the battery system, have yet to be thoroughly elucidated.

Here, we elucidate the origin and mechanism of phase transition and degradation in TMS (CuS, Cu_2_S, FeS, MnS, CoS_2_, and Ni_3_S_2_) anodes, which exhibit extraordinary electrochemical behavior. During the initial sodiation/desodiation of bulk TMS, NaPSs are generated and subsequently react with the Cu‐CC in the later cycles, forming NaCu_5_S_3_ and Cu_2_S phases on the surface. These interfacial reaction products participate in Na^+^ storage and trigger progressive phase transitions toward Cu*_x_* S*_y_* in the following cycles. Furthermore, we revealed that, after prolonged cycling, the crystal structure of all TMS anodes is ultimately transformed into nanograined Cu_1.8_S, irrespective of their initial states. In addition, we conduct a comprehensive investigation of the deactivation and redeposition behavior of the original transition metals in TMSs, which is directly correlated with the stability of battery system. Furthermore, the factors influence the kinetics of irreversible conversion reactions and phase transformation of TMS are systematically studied. This study investigates similarity in crystal structures between Na_2_S and Cu_1.8_S drives the phase transition of TMS anodes into the Cu_1.8_S phase during electrochemical cycling. This stabilized phase exhibited stable operation with the Al‐CC, whereas other TMSs experience capacity degradation or failure.

These results are anticipated to provide a comprehensive perspective on the TMS phase evolution under operation, offering practical guidelines for the configuration of robust TMS‐based sodium‐ion battery anodes. For the investigation, we utilized crystal structural characterization employing transmission electron microscopy (TEM) and X‐ray diffraction (XRD) in combination with electrochemical characterization and density functional theory calculations (DFT).

### 1.1 Electrochemical Characterization of Bulk Transition Metal Sulfide Anodes with Copper Current Collector

Transition metal sulfide (TMS) anodes, which store sodium ions via conversion reactions, are widely acknowledged for exhibiting optimal functionality with the ether‐based electrolytes [20, 21]. However, the inevitably occurring irreversible conversion reactions during repeated charge/discharge cycles generate NaPSs, which readily interact with the copper current collector (Cu‐CC) forming Cu‐incorporated phases.

Therefore, to investigate the effect on Cu‐CC in electrochemical performance of TMS anodes, we prepared various types of bulk TMS anodes, including FeS, MnS, CoS_2_, Ni_3_S_2_, Cu_2_S, and CuS (Figures S1–S4). All the TMS anodes were discharged and charged repeatedly with a voltage range from 0.05 V to 2.6 V in a half‐cell system with the current density of 0.5 A g^−1^. During the execution of half‐cell tests employing an ether‐based electrolyte (1 M NaPF_6_ in diglyme) and a Cu‐CC, it was confirmed that all bulk TMSs are operated reliably without failure or severe degradation, even in the absence of detailed modifications, such as nanostructure design, carbon decoration, and geometrical optimization, while utilizing carbonate‐based electrolyte resulted in severe cell degradation and failure (Figure S5a). This tendency, as observed in carbonate‐based electrolytes, is consistent with the findings of previous studies [22, 23]. TMS‐based anodes have been observed to demonstrate poor cycle performance in the presence of carbonate‐based electrolytes (Figure S5b). This phenomenon originates from undesirable side reactions between polysulfides and carbonate solvents, as well as the dissolution of sulfide species [24, 25]. Therefore, all electrochemical tests in this study were conducted using ether‐based electrolytes.

**FIGURE 1:**
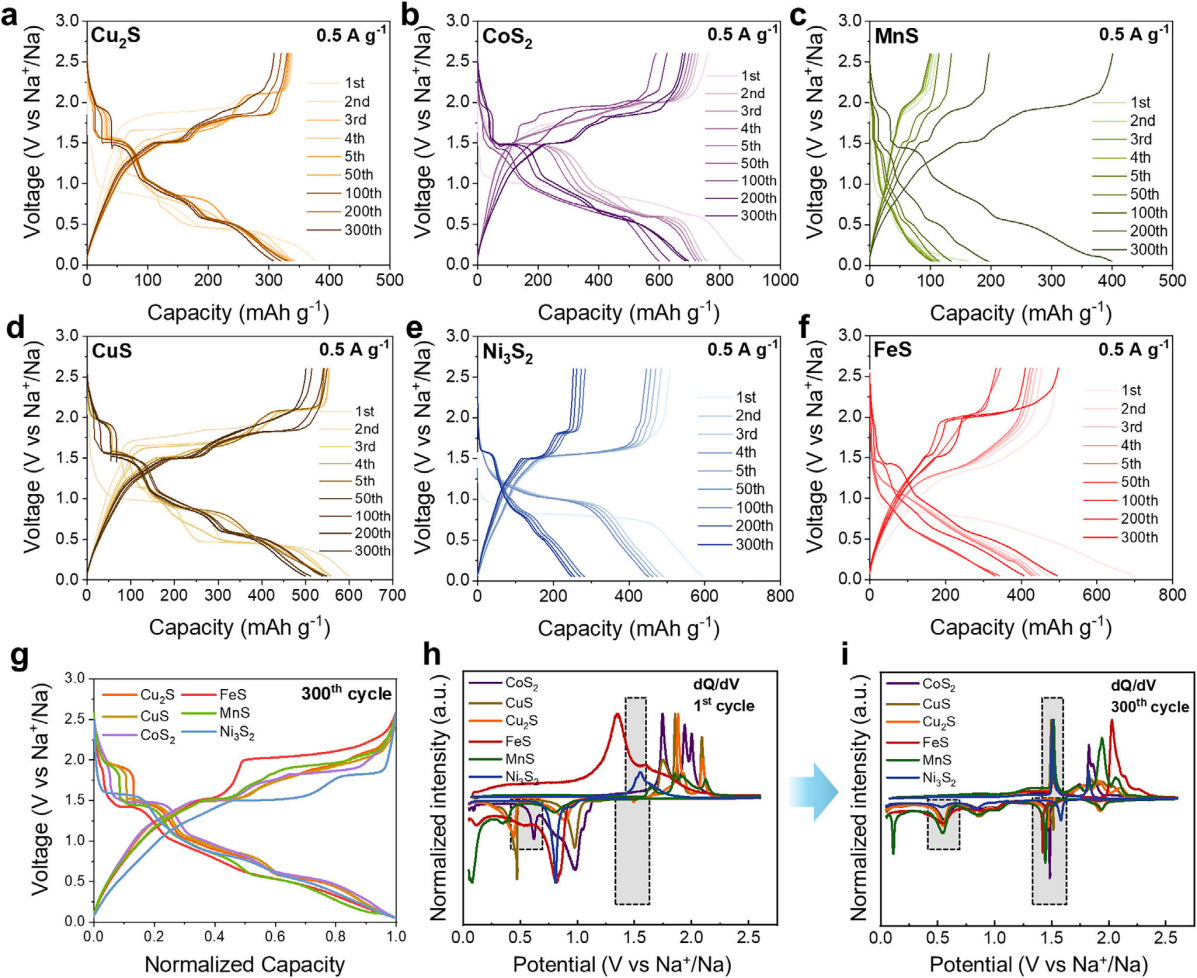
Electrochemical characterization of bulk TMS anodes during 300 cycles. (a‐f) Charge–discharge profiles of bulk TMS anodes. (g) Normalized and merged charge–discharge profiles of bulk TMS anodes. (h) dQ/dV plots of bulk TMS anodes at the first cycle. (i) dQ/dV plots of bulk TMS anodes at the 300th cycle.

In the initial cycles, each TMS anodes exhibited a distinguishable charge‐discharge profile, which represent their intrinsic sodiation/desodiation pathway and redox reactions (Figure 1a–f; Figure S6). However, when the cycles proceeds, their charge‐discharge profiles were changed and converged to the similar shapes (Figure 1g; Figure S6). The dQ/dV plot of each TMS anode also shows convergence of cathodic and anodic peaks into analogous peak positions upon charge/discharge cycles (Figure 1h–i). Specifically, all TMSs exhibited analogous dQ/dV peaks at approximately 1.51 V for desodiation, and 0.55 and 1.45 V for sodiation (gray region marked by a black dashed line in Figure 1i. This finding suggests that the electrochemical reactions that occurs in TMS anodes are consistent in the prolonged cycles.

### 1.2 Phase Transition in Transition Metal Sulfide Anodes Upon Cycling

**FIGURE 2:**
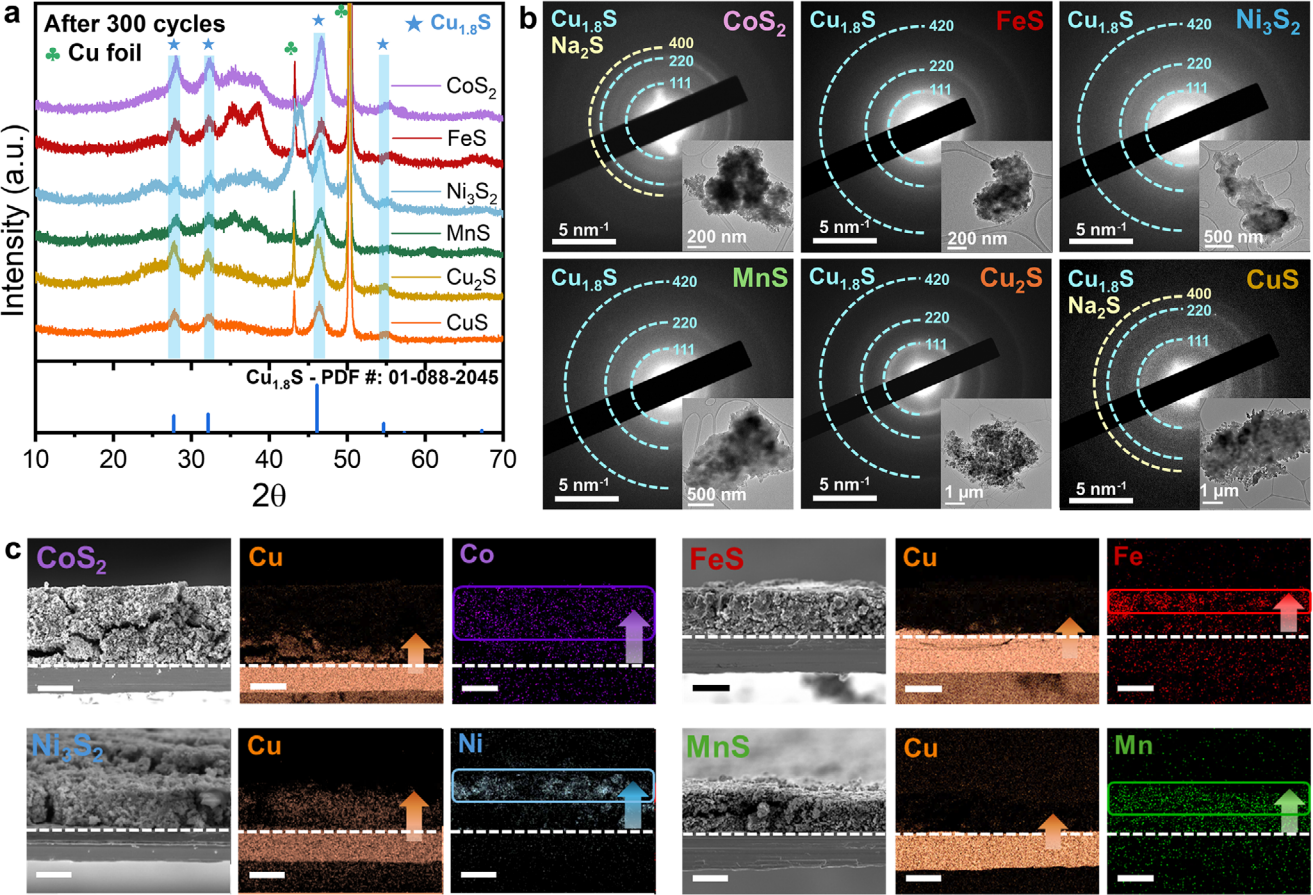
(a) Ex situ XRD results of bulk TMS anodes after 300 cycles. (b) SAED patterns and corresponding bright‐field TEM images of bulk TMS anodes after 300 cycles. (c) Cross‐sectional SEM‐EDS elemental mapping results of bulk TMS anodes after 50 cycles (scale bar, 20 µm).

According to the electrochemical characterization (e.g., charge/discharge profiles, and dQ/dV peak analysis) results, the redox reactions of the TMS anodes during sodiation/desodiation gradually follow the same reaction pathway, indicating that a phase transition has occurred at the TMS electrodes upon cycling. Therefore, to verify the phase transition and to identify the final crystal structure and morphological features (e.g., grain size and morphology) of the TMS anodes after 300 cycles, ex situ X‐ray diffraction (XRD) and ex situ transmission electron microscopy (TEM) analyses were performed. Notably, the crystal structure of all TMS anodes was transformed into Cu_1.8_S without exception (Figure 2a). The selected‐area electron diffraction (SAED) patterns and high‐resolution TEM (HR‐TEM) images of each TMS further confirmed the consistent morphological evolution from bulk TMS particles to nanograined Cu_1.8_S (Figure 2b; Figure S7).

To further investigate how the phase transition behavior progresses upon cycles, a scanning electron microscope with energy‐dispersive X‐ray spectroscopy (SEM‐EDS) analysis was performed on both surface and cross‐section of each electrode after 50th and 300th cycles.

After 50 cycles, elemental mapping results indicate that the Cu‐incorporated phase transition begins at the interface between the active material and the Cu‐CC (Figure 2c; Figure S8a). Interestingly, the existing original TMs exhibited a phenomenon in which they appeared to be propelled toward the electrode surface (Figures S8b and S9a). After 300 cycles, Cu was found to be distributed over most of the electrode, and the original transition metal was confirmed to have either disappeared or become isolated on the surface (Figure S9b,c). Since the observed Cu must have originated from the CC, there should be the factor that triggering Cu‐incorporated phase transition. When the cells were disassembled after 300 cycles, the separators exhibited a black or deep orange coloration, indicating that the NaPS shuttling effect had prevailed throughout the 300 cycles (Figure S10) [8].

### 1.3 Effect of Sodium Polysulfides in Phase Transition of Transition Metal Sulfide Anodes

Sodium storage through conversion reactions of TMS is often accompanied by low coulombic efficiency, particularly in the initial cycles. The underlying causes can be attributed to various factors, including the formation of the SEI layer [26, 27], pulverization of active materials [28, 29], and sodium entrapments in the active materials as a form of Na_x_TM_y_S_z_ [30, 31, 32]. Those phenomena induce irreversible sodiation/desodiation reactions, during which sodium polysulfides are likely to be formed.

**FIGURE 3:**
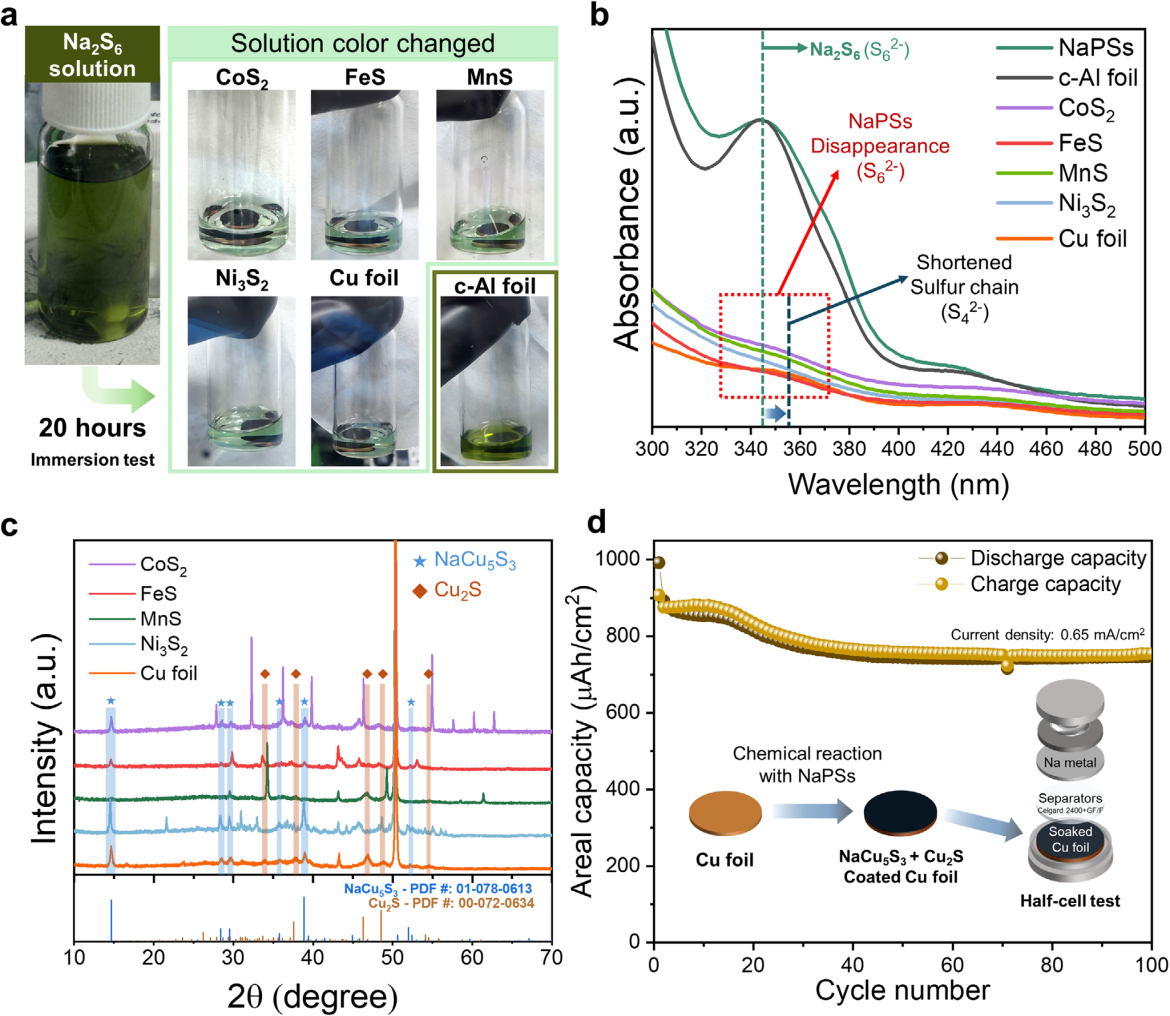
(a) Optical images of the pristine NaPS solution and the solutions obtained after 20 h of immersion. (b) UV‐vis spectra of the pristine NaPS solution and the solutions collected after the immersion test. (c) Ex situ XRD patterns of the TMS electrodes and Cu‐CC after the immersion test. (d) Cycling performance of the reaction‐products‐coated Cu‐CC and a schematic illustration of the half‐cell configuration.

Therefore, aforementioned phase transition to Cu_1.8_S is presumed to be closely related with the reaction between the Cu‐CC and NaPSs. Accordingly, an immersion test was conducted on the TMS electrodes, bare Cu foil, and carbon‐coated aluminum (c‐Al) foil to clarify the effect of NaPSs on the Cu‐CC and active materials. Since Na_2_S_6_ is the predominant NaPS species produced during the irreversible conversion reaction process [33, 34, 35], Na_2_S_6_ solution was employed in the immersion test (Figure S11a). After immersing electrodes in the as prepared 0.001 M Na_2_S_6_ solution for 20 h, we noticed that except for the c‐Al foil case, all of the NaPS solutions experienced color changes (Figure 3a). The color changes observed in the solution reflect alterations in the chemical structure of the NaPSs. To further probe these chemical structural variations after the reactions, UV–vis spectroscopy was performed on the remaining solutions (Figure 3b). The disappearance of the peak at ∼345 nm, assigned to Na_2_S_6_ (${S_{6}}^{2-}$), was observed for both the Cu foil and TMS electrodes, while a new, low‐intensity peak appeared at ∼356 nm in the higher‐wavelength region [33]. These spectral changes indicate that most of the Na_2_S_6_ is consumed through chemical reactions, resulting in a shortening of the sulfur chain to ${S_{4}}^{2-}$. This suggests that the sulfur in Na_2_S_6_ predominantly participates in these reactions.

To verify the reaction products and effect of NaPSs on active materials, ex situ XRD was performed (Figure 3c; Figure S11b). Interestingly, after the chemical reaction of NaPSs with Cu foil and TMS electrodes, the NaCu_5_S_3_, and Cu_2_S phases were produced in common without any evolution of the sodiated intermediate phases of TMSs. The NaPSs demonstrated no reaction with the active materials; rather, they underwent a chemical reaction only with the Cu foil. The intrinsic porous structure of TMS electrodes allows NaPSs to permeate and react with the surface of the Cu foil.

In order to investigate the influence of these reaction products on TMS anodes’ electrochemical performances, we assembled the coin cell with the reaction products‐coated Cu‐foil and conducted half‐cell test (Figure 3d). Although there were no conductive agents and binder, the assembled cell exhibited reliable cycle performance. In addition, after multiple discharge‐charge cycles, the reaction products also experienced phase transition to Cu_1.8_S (Figure S12). This result implies that the NaPSs formed in the initial cycles interact with the Cu‐CC, become retrapped, and thereby contribute to sodium storage in subsequent cycles.

NaPSs formation may persist throughout subsequent charge–discharge cycles, but for bulk‐scale TMS anodes, a relatively large amount is expected to form during the initial cycles due to their limited active surface area and the long distance path required for Na^+^ diffusion [4, 30, 31, 36].

Compared to bulk TMS anodes, nanoparticle TMSs (NP‐FeS, NP‐CoS_2_) exhibited higher coulombic efficiency, which can be attributed to their smaller grain size (Figures S13–S15) that facilitates reversible conversion reactions and suppresses excessive formation of sodium polysulfides (NaPSs) [25, 37]. In NP‐TMSs, the phase transition to Cu_1.8_S (Figure S15h–i) occurred more slowly than in their bulk counterparts, as shown by charge–discharge profiles (Figure S15a–f). This suggests that while the fundamental phase transition remains the same, it could be kinetically moderated by the nanoscale structural effects. These findings support the idea that NaPSs are more frequently generated during the initial cycles in bulk TMS anodes due to the limited reversibility of conversion reactions, which is a consequence of their large grain size. Sodium storage through the conversion reaction becomes more efficient as the bulk particles break into smaller domains (nanograins) during repeated charge‐discharge cycles [25, 38]. However, the formation of NaPSs and the subsequent phase transition to Cu_1.8_S remain inevitable in the TMS anode with the Cu‐CC system.

### 1.4 Transition Metal Deactivation Phenomena and Redeposition on the Counter Electrode

**FIGURE 4:**
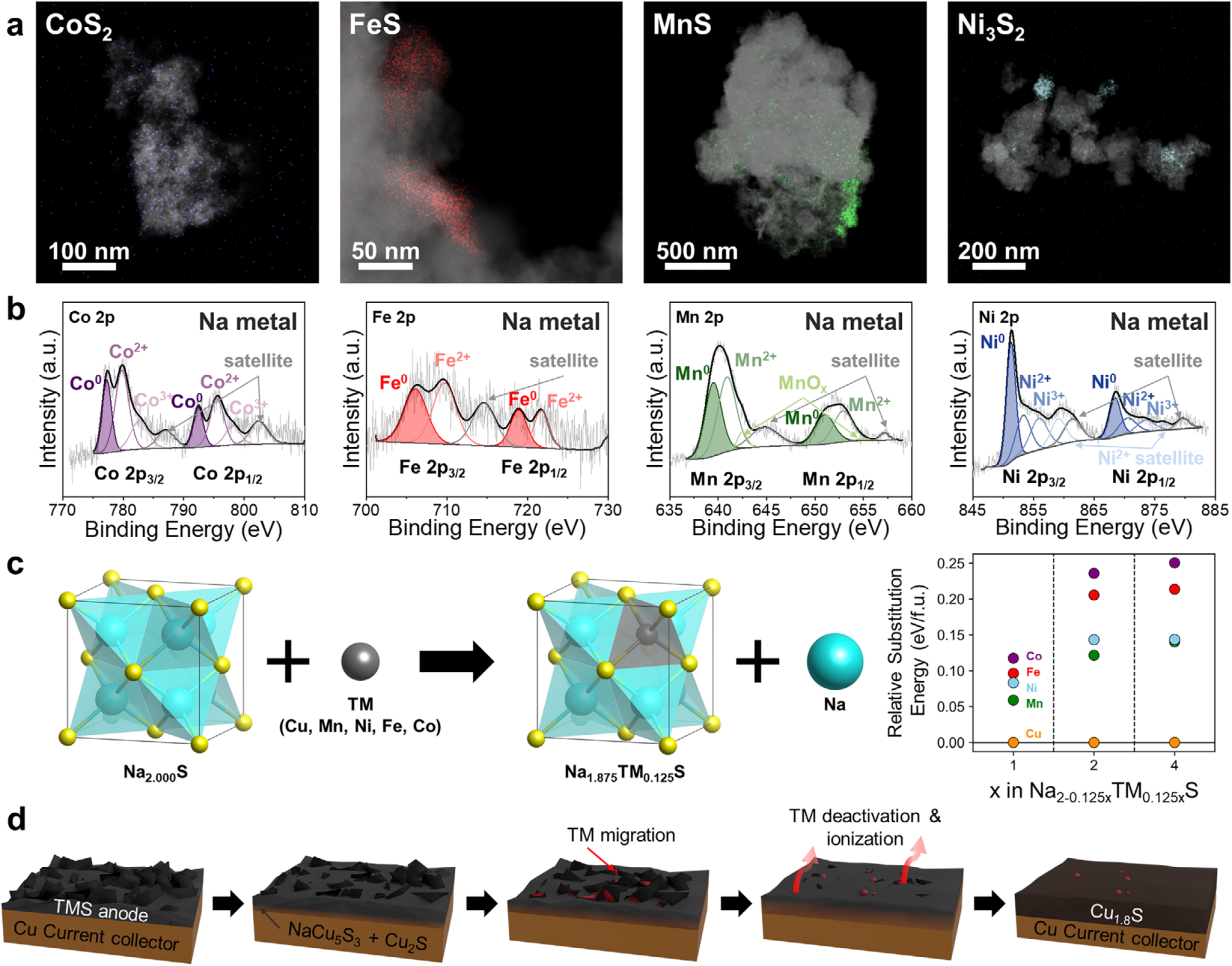
(a) STEM‐EDS colormix images combining HAADF and TM elemental maps of CoS_2_, FeS, MnS, and Ni_3_S_2_ electrodes after 300 cycles. (b) XPS fitting data for each TM element at the counter electrode. (c) Relative formation energy calculation data of substituting Na^+^ through TM within Na_2_S frame. (d) Schematic illustration of the Cu‐induced phase transition and degradation of TMS anodes.

We further noticed that after 300 cycles, although Cu was predominantly detected on the overall electrode, the original TM was infrequently detected as a localized region in the electrodes along with its particularly low compositional ratio (Figure 4a; Figures S16–S19). To verify the phase of leftover TM elements, we conducted scanning transmission electron microscopy–energy‐dispersive X‐ray spectroscopy (STEM‐EDS), and HR‐TEM analysis of each electrode after 300 cycles. As a result, the original TM within the cycled electrodes existed in metal nanoparticles, indicating its deactivation during subsequent cycles (Figures S20–S23). Ex situ X‐ray photoelectron spectroscopy (XPS) was also performed on bulk TMS electrodes before and after 300 cycles. As confirmed by STEM‐EDS and HR‐TEM analyses, the original TM species were observed to remain on the electrode in both metallic and partially ionized states (Figure S24). Meanwhile, the chemical state of Cu in all electrodes was identical to that of Cu_1.8_S (Figure S25).

The markedly reduced content of the original TM suggests substantial loss from the electrode, likely through ionization and subsequent dissolution into the electrolyte. These partially dissolved TM species can migrate across the separator and reach the counter electrode. To examine their impact on the cell, ex situ XPS and SEM‐EDS analyses were conducted on the Na counter electrode (Figure 4b; Figures S26 and S27). The results reveal a homogeneous distribution of TM on the Na surface, predominantly in a metallic state with a minor portion remaining partially ionized. Owing to their small ionic radius, ionized TM species can readily traverse the separator and undergo reduction reactions upon contact with the counter electrode, leading to their redeposition as metallic TM. Metallic TM nanoparticles have been observed to function as catalysts, which may result in undesirable consequences such as electrolyte decomposition, interface instability, and reaction heterogeneity [39, 40, 41]. In full‐cell configurations, the accumulation of ionized/metallic TM on the cathode surface is expected to increase interfacial resistance and thereby deteriorate the overall cell performance.

### 1.5 Origins of the Copper‐induced Phase Transition During the Desodiation Process

When the phase transition to Cu‐incorporated sulfides commences in the subsequential cycle, both the Cu and the original TM coexist within the electrode. However, it should be noted that the existing TM undergoes deactivation and ionization as the cycle progresses, with the redox reactions being predominantly governed by Cu. To date, many researchers have employed the hard soft acid base (HSAB) theory to explain the cause of this phase transition to Cu*_x_* S. However, it is difficult to explain those aforementioned phenomena solely through HSAB theory adequately. Consequently, a more thorough examination of this phenomenon is required.

The Cu‐incorporated phase transition can occur during either the sodiation or desodiation process. However, given that the final reaction product of conversion reaction at the fully sodiated states results in Cu or TM existing as metallic nanoparticles [42, 43, 44, 45], the assumption of a phase transition occurring during sodiation process appears logically untenable. Therefore, we suspect that Cu‐induced phase transition primarily takes place during the desodiation process.

A notable and common characteristic of TMS anodes is the absence of specific plateaus in the charge‐discharge profile during initial desodiation process while capacity was measured and the potential increased at the same time (Figure 1a–f). Therefore, it can be postulated that initial desodiation is accompanied with solid‐solution‐like reactions.

At the initial stage of desodiation, Na_2_S is likely to undergo partial sodium extraction, forming a Na_2‐_ *_x_* S type intermediate. This sodium‐deficient phase is inherently metastable due to charge imbalance and therefore requires compensation. One possible pathway involves partial sulfur oxidation accompanied by sodium polysulfide formation, while another involves conversion reactions coupled with oxidation of the TM nanoparticles. In TMS electrodes, these two pathways may coexist; however, not all Na_2_S is transformed into polysulfides. Instead, locally oxidized TM species generated at the onset of desodiation may participate in charge compensation of the Na_2‐_ *_x_* S intermediate.

Based on this assumption, to elucidate why Cu participates in the initial desodiation process, the relative substitution energies of TM ions in the cubic Na_2_S structure were calculated using DFT calculations (Figure 4c). The results show that Cu is the most favorable TM species for substituting Na during desodiation across various concentrations (x = 1, 2, and 4).

In summary, the NaPS generated during the initial cycle reacts with the Cu foil to form NaCu_5_S_3_ and Cu_2_S on the surface. During the following cycles, as these reaction products begin to participate in the actual sodium storage reactions, a Cu‐incorporated phase transition is initiated at the interface. The incorporated Cu then replaces Na in Na_2_S during desodiation, a process that is energetically more favorable than the participation of the original TM, thereby transforming the initial TMS into a Cu‐incorporated sulfide (Figure 4d).

### 1.6 Crystal Structural Relation with Final Structure and Saturated Sodium Storage

**FIGURE 5:**
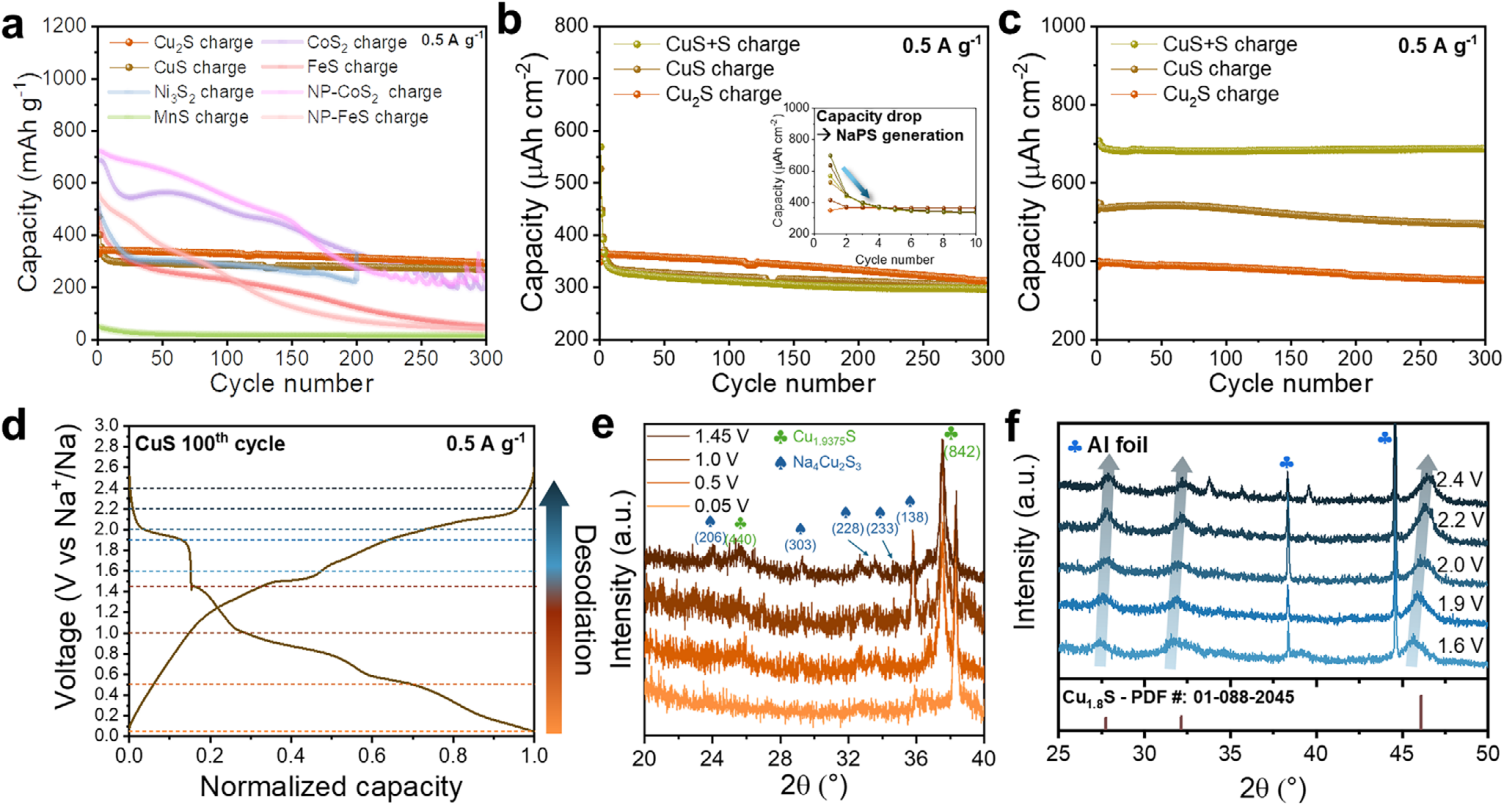
(a) Cyclic performance of TMS anodes on the c‐Al current collector. (b) Specific areal capacity of Cu‐S anodes on the c‐Al current collector. (c) Specific areal capacity of Cu‐S anodes on the Cu current collector. (d) Charge‐discharge profile of CuS on the c‐Al current collector at the 100th cycle. (e,f) Ex situ XRD patterns of CuS during the 100th desodiation.

We confirmed that all TMS anodes are experiencing a phase transition to Cu_1.8_S when Cu foil is used as the CC. However, to prevent such CC corrosion and to exploit the advantages of SIBs that allows the use of Al as the anode‐side CC, the compatibility of TMSs with the Al‐CC also should be verified. Accordingly, we conducted half‐cell tests under the same cycling conditions as those used for Cu‐CC, employing carbon‐coated aluminum current collector (c‐Al‐CC). We found that, except for Cu‐based sulfides (CuS and Cu_2_S), all bulk TMS anodes (FeS, MnS, CoS_2_, and Ni_3_S_2_) exhibited failure or severe degradation during cycling, even when nanoparticle modifications (NP‐FeS and NP‐CoS_2_) were applied (Figure 5a). These degradations arise from the irreversibility of the conversion reactions [25, 46], which lead to the significant formation of NaPSs. The underlying reason for the reliability of bulk TMS electrodes in the Cu‐CC system is the reutilization of NaPSs in the sodium storage process, facilitated by the Cu supplied from the CC.

Notably, the capacity of CuS and Cu_2_S with c‐Al‐CC converged to a similar value, which is not observed tendency in the Cu‐CC system. Therefore, to investigate the influence of the Cu to S ratio on the final phase evolution in the absence of external Cu supply from the current collector, and to elucidate the origin of the stabilized Cu_1.8_S phase among Cu*_x_* S materials, electrodes with three different stoichiometries (Cu_2_S, CuS, and CuS+S) were fabricated on c‐Al‐CC and evaluated in half cells (Figure 5b; Figure S28). Based on the loading mass of active materials (∼1 mg/cm^2^), the calculated theoretical areal capacity of each electrode is 336.7 µAh/cm^2^ for Cu_2_S, 560.6 µAh/cm^2^ for CuS, and 839.6 µAh/cm^2^ for CuS+S, respectively. However, the areal capacity of each electrode was saturated to almost the same value (∼300 µAh/cm^2^), not to the expected capacities of each electrode. Specifically in the initial cycles, S‐rich electrodes (CuS and CuS+S) experienced significant capacity decay.

In contrast, when employing Cu‐CC, the electrodes exhibited minimal capacity drop in the initial cycles, and showed stable operation upon 300 cycles with a capacity similar to the estimated value (Figure 5c). This phenomenon could be postulated that these NaPS intermediates appear to be effectively confined within the electrode due to the high mass ratio (20 wt%) of conductive agent with a large specific surface area and applying a polyacrylic acid (PAA) binder containing a carboxyl group, which effectively anchors NaPSs via van der Waals adsorption (Figure S29) [47, 48]. This trapping facilitates the reutilization of the deactivated sulfur (dissolved as a form of NaPSs) in subsequent redox processes, resulting in an enhanced capacity compared with the c‐Al‐CC case.

The ex situ XRD results of the three electrodes paired with c‐Al‐CC and with Cu‐CC after 300 cycles indicate that the Cu_1.8_S phase is consistently formed as the final structure, regardless of the electrode type (Figure S30). Notably, repeated charge–discharge cycling reproducibly leads to the formation of Cu_1.8_S, independent of variations in the current‐collector material or the Cu‐S stoichiometric ratio.

In order to ascertain the mechanism by which the final crystalline structure invariably converged to Cu_1.8_S, it is necessary to examine the crystal structural changes during desodiation. Therefore, we performed ex situ XRD analysis of CuS electrodes as a representative case with c‐Al‐CC to avoid artifacts such as the interaction between NaPSs and Cu‐CC. The ex situ XRD analysis on the same electrodes at various voltage levels (0.05, 0.5, 1.0, 1.45, 1.6, 1.9, 2.0, 2.2, and 2.4 V) from the 100th charge (desodiation) process was performed (Figure 5d).

When fully discharged state (0.05 V), the electrode exhibited cubic phase of Na_2_S (Figure S31). As desodiation commenced, the Na_4_Cu_2_S_3_ (PDF: 01‐081‐0343) was observed to gradually evolve up to 1.45 V along with the evolution of Cu_1.9375_S (Djurleite, PDF: 01‐071‐1383) phases (Figure 5e). Despite approximately 50% of the sodium remaining within the electrode at 1.6 V, it can be observed that the XRD peak positions of the electrode resemble those of Cu_1.8_S. Notably, as desodiation progresses, each main diffraction peak undergoes a shift toward higher angles, indicating a reduction in lattice structure size and d‐spacing while maintaining its structural basis of Cu_1.8_S. This phenomenon is attributable to the smaller ionic radius of Cu relative to Na (Figure 5f).

To elucidate this distinctive phase‐transition phenomenon during desodiation, we examined the crystal structures of the three phases (Na_2_S, Na_4_Cu_2_S_3_, and Cu_1.8_S). Interestingly, all three phases exhibit remarkably similar sulfur atomic arrangements, with comparable S–S distances (Figure S32). Therefore, it can be postulated that the phase transition proceeds along a kinetically favorable pathway facilitated by the structural similarity among the phases. In addition, Cu_1.8_S exhibits higher electrical conductivity compared to allotropes of Cu_2_S due to the copper vacancy within the unit cell [49]. Additionally, Cu_1.8_S demonstrates high copper diffusivity, thereby emphasizing that the phase transition toward Cu_1.8_S is kinetically favorable [50].

When Cu foil is used as the CC in TMS anodes, additional Cu can be supplied from the CC through chemical reactions driven by the generated NaPSs, ultimately forming Cu_1.8_S. Under these conditions, the cell capacity is determined by the total sulfur content of the active materials. Conversely, with an Al‐CC system, the capacity is not governed by the sulfur content but is instead restricted by the stoichiometric ratio between Cu and S. In sulfur‐rich conditions, such as CuS or CuS+S, the excess sulfur cannot participate in the reversible conversion reaction and dissolves into the electrolyte as NaPSs, and causes capacity degradation until the system meets the structural and stoichiometric requirements for the Cu_1.8_S, consistent with the saturated capacity.

When the TMS‐based battery primarily operates through the Na‐Cu‐S based reaction pathway, in which Cu is supplied by the Cu‐CC, an increase in electrode loading mass results in a greater extent of TM ionization and promotes the formation of NaPSs. These processes are expected to substantially compromise the overall stability of the battery. In particular, the depletion of Cu in the Cu‐CC induces significant structural degradation of the CC, ultimately leading to cell failure. The results of this study suggest that the electrochemical reaction pathway of the TMS anode is constrained by the stoichiometric ratio of Cu:S in the electrode system, including the CC. Deviations from this ratio invariably lead to capacity loss due to irreversible reactions.

The most critical factor in mitigating the deactivation, dissolution, and redeposition of TM species is achieving highly reversible conversion reactions in TMS anodes, thereby effectively suppressing the generation of NaPSs. Strategies developed to enable reversible conversion reactions in TMS based SIBs, such as nanoparticle engineering [51], carbon hybridization [52], and electrolyte optimization [53], can be applied to mitigate TM dissolution in both Cu‐CC and Al‐CC systems. Structural refinement and carbon‐based hybridization promote homogeneous reaction kinetics and suppress irreversible reactions. In addition, electrolyte optimization may limit TM solubility and migration. Together, these approaches offer an effective pathway to stabilize TMS electrodes in Cu‐CC systems and to achieve stable operation in Al‐CC systems without relying on external Cu supply.

## 2 Conclusions

We investigated the exact phase transition mechanism of TMS anodes, which is triggered by NaPSs and Cu‐CC, and how this phase transition propagates. The initial sodiation and desodiation process accompanying irreversible conversion reactions, thereby generates NaPSs within the cell system. It chemically reacts with the Cu‐CC and forms NaCu_5_S_3_ and Cu_2_S phases on the surface in the subsequent cycles.

The Cu supplied in this manner continues to migrate into the active materials of the electrode in subsequent cycles. When desodiation commences in the fully discharged (sodiated) state, Cu preferencially substitutes sodium in Na_2_S, that has been extracted in an energetically stable manner. Therefore the existing original transition metal is deactivated in the electrodes and dissolved within the electrolyte as a form of ionized state, resulting in metallic redeposition on the counter electrode which could cause side‐reactions and short‐circuit problems.

Furthermore, we investigated a crystal structure related phase transition mechanism in TMS anodes, suggesting that Cu_1.8_S represents an energetically and kinetically favorable phase in conversion reaction‐based SIB systems. These results indicate that Cu_1.8_S may serve as a promising TMS for Al‐CC based configurations, with the potential to enhance cell lifetime and battery level energy density. Our findings are expected to provide important insights for the future practical application of TMS‐based SIB system.

## Conflicts of Interest

The authors declare no conflict of interest.

## Supporting information




**Supporting File**: advs74061‐sup‐0001‐SuppMat.docx.

## Data Availability

The data that support the findings of this study are available from the corresponding author upon reasonable request.
