# Supplementary material for: Unveiling Copper‐Induced Phase Transitions and Degradation Mechanisms of Transition Metal Sulfide Anodes for Sodium‐Ion Batteries
Source: Adv Sci (Weinh). 2026 Jan 28;13(19):e24191. doi: 10.1002/advs.202524191 (PMC13045226; doi:10.1002/advs.202524191)
Supplement: Supplementary file 1 — Supporting File: advs74061‐sup‐0001‐SuppMat.docx. [file ADVS-13-e24191-s001.docx]

**Supporting Information**

**Unveiling Copper-Induced Phase Transitions and Degradation Mechanisms of Transition Metal Sulfide Anodes for Sodium-Ion Batteries**

*Jacob Choe^1‡^, Junpyo Hur^1‡^, Sanghyeon Park^1,2^, Jeongmin Kim^1^, Jaeeun Joo^1^, Jae Yeol Park^,2^, Chan-Woo Lee^2^ and Jong Min Yuk^1^**

^1^ Department of Materials Science & Engineering, Korea Advanced Institute of Science and Technology (KAIST), 291 Daehak-ro, Yuseong-gu, Daejeon, 34141, Republic of Korea.

^2^ Energy Storage Research Department, Korea Institute of Energy Research (KIER), 102 Gajeong-ro, Yuseong-gu, Daejeon, 34113, Republic of Korea.

*^‡^*: These authors contributed equally.

*: Correspondence to this author: [jongmin.yuk@kaist.ac.kr](mailto:jongmin.yuk@kaist.ac.kr)

**METHODS**

**Transition metal sulfide electrode preparation**

For bulk transition metal sulfides, Nickel Sulfide (Ni_3_S_2_, Sigma-Aldrich), Manganese (II) Sulfide (MnS, Sigma-Aldrich), Copper (I) Sulfide (Cu_2_S, Sigma-Aldrich), Copper (II) Sulfide (CuS, Sigma-Aldrich), Iron (II) Sulfide (FeS, Sigma-Aldrich), and Cobalt (IV) Sulfide (CoS_2_, Sigma-Aldrich) were purchased and used without further purification. For nanoparticle metal sulfides, bulk metal sulfides were ball-milled 5 hours with 400 rpm by using 3D ball milling. The weight ratio between active material and zirconia ball was set as 1:10.

The fabrication of transition metal sulfide electrodes was carried out by combining 70 wt% active material, 10 wt% poly (acrylic acid) (PAA, Sigma-Aldrich), and 20 wt% carbon black (acetylene, 100% compressed, Alfa Aesar) with deionized water to make a slurry. The slurry was subsequently coated onto the current collector using an automatic applicator. Either Cu foil (Wellcos Corporation) or conductive carbon-coated Al (c-Al) foil (Wellcos Corporation) was used as a current collector. The cast slurries were dried in a vacuum oven at 80 °C for 8 hours. The loading mass of the active material was ~ 1.5 mg/cm^2^.

To investigate the intrinsic phase transition mechanism while excluding external Cu contributions from the current collector, electrodes with different Cu:S stoichiometries (2:1, 1:1, and 1:2) were fabricated on a c-Al current collector with a mass loading of 1 mg/cm^2^. To simulate a Cu:S stoichiometry of 1:2, bulk CuS and sulfur were homogenized using a 3D ball mill at 400 rpm for 4 hours with a molar ratio of 1:1. The weight ratio of active material to zirconia milling balls was maintained at 1:10.

**Electrochemical cell tests**

A CR2032-type coin cell was used in the electrochemical cell test. All coin cells were assembled in a glove box under an argon atmosphere. Galvanostatic cell tests were performed with a battery cycling system (WBCS 3000L, Wonatech). All the electrochemical tests were performed under a current density of 0.5 A g^-1^, and at a constant temperature of 25 °C. Both Celgard 2400 (PP, Celgard) and glass-fiber filter (GF/F, Whatman) were used as separators. Sodium hexafluorophosphate (NaPF_6_, 1 M, Sigma‒Aldrich) in diglyme (Diethylene glycol dimethyl ether, Sigma-Aldrich) was used as the electrolyte. Pure Na foil (Sigma‒Aldrich) was used as the counter electrode for the half-cell test.

**Ex-situ crystal structure analyses**

The cycled coin cells were disassembled in an argon-filled glove box. The electrode was washed with diglyme to remove residual electrolyte and salts, and dried overnight for further analyses. The X-ray diffractometer (XRD, SmartLab, Rigaku) was utilized to confirm the change of the crystal structure in the electrode state.

The selected area electron diffraction (SAED) pattern of the electrode was obtained through Transmission Electron Microscopy (TEM). Pristine and cycled transition metal sulfide electrodes were sonicated and dispersed in diglyme. The dispersed electrode particles were drop casted onto a lacey carbon film and carbon film-supported Au grid (300 mesh, EMS). A direct-detect type camera (One View, Gatan) equipped TEM (JEM-2100F, JEOL) was used for SAED acquisition. For high resolution TEM imaging and Scanning Transmission Electron Microscopy-Energy Dispersive Spectroscopy (STEM-EDS) were conducted with 4 windowless SDD EDS system (Super X, Thermo Fisher Scientific) equipped TEM (Talos F200X G2, Thermo Fisher Scientific)

**Surface and cross-section analysis**

The surface and cross-sectional morphology of the transition metal sulfide anode was examined with field emission Scanning Electron Microscope (SEM, SU5000, Hitachi). For pristine and cycled electrodes, energy dispersive spectroscopy (EDS) analysis of the surface and cross-sections was conducted with a SEM (SEM-EDS) to confirm the copper diffusion phenomenon.

**Elemental analysis**

To determine the element distribution of cycled transition metal sulfide anodes, EDS analysis of the cycled electrode was conducted with Scanning Transmission Electron Microscopy (STEM-EDS, Talos F200X, Thermofisher). X-ray Photoelectron Spectroscopy (XPS, K-Alpha+, Thermo Fisher Scientific) was conducted with Vacuum Transfer Module (Thermo Fisher Scientific) to determine the oxidation state of each transition metal of the electrodes and sodium metal after cycling.

**Sodium polysulfide soaking method**

0.001M of Na_2_S_6_ solution in diglyme was prepared to simulate the solution in which polysulfides are generated during initial cycles. Na_2_S_6_ solution was prepared by stirring sodium sulfide (Na_2_S, Sigma-Aldrich) and sulfur (S, Sigma-Aldrich) in a stoichiometric ratio with diglyme. Transition metal sulfide electrodes, Cu foil, and carbon-coated Al foil were slitted to 14Φ, soaked with 4 ml of prepared Na_2_S_6_ solution, and immersed for 20 hours in an argon-filled glove box. All electrodes and bare foils were rinsed with diglyme after soaking to remove the remaining sodium polysulfides on the surface and dried overnight. For the soaked Cu foil, the electrochemical cell test was conducted under a current density of 0.65 mA/cm^2^ following the assembly of the coin cell.

***Ex Situ* UV–vis Measurements**

UV–vis spectra for the reference solutions and the *ex situ* study were obtained on a 2-channel spectrometer Lambda 1050 (Perkin Elmer) using argon-filled, sealed quartz glass cuvettes (Hellma Analytics, QS115).

**Computational details**

DFT calculations were performed using the Vienna ab initio Simulation Package (VASP)^[1]^ based on the projector-augmented wave (PAW) method and the Perdew–Burke–Ernzerhof (PBE)^[2]^ generalized gradient approximation. A plane-wave basis for wave functions was employed with an energy cutoff of 500 eV. The 3 × 3 × 3 k-point grid was used to sample the Brillouin-zone using Monkhorst–Pack method.^[3]^ The convergence criteria were set to 1.0 × 10⁻⁶ eV and 0.02 eV/Å for the electronic and ionic steps, respectively. The substitution energy (*E*_Sub_) was calculated with following equation:

$$E_{\mathrm{Sub}}=(E_{\mathrm{Na}_{2-x}\mathrm{TM}_{x}S}+xE_{\mathrm{Na}})-\left( E_{\mathrm{Na}_{2}S}+xE_{\mathrm{TM}} \right) [eV/f.u.]$$

where $E_{\mathrm{Na}_{2-x}\mathrm{TM}_{x}S}$, $E_{\mathrm{Na}_{2}S}$, $E_{\mathrm{Na}}$, and $E_{\mathrm{TM}}$are the energies of transition metal substituted and pristine Na_2_S, Na metal and transition metal (Cu, Co, Fe, Mn, and Ni). The crystal structures of Na and Fe are body-centered cubic, whereas those of Cu, Co and Ni are face-centered cubic. The energy of Mn metal was calculated using α-Mn phase (cubic, space group I-43m). The VESTA software was used to visualize the crystal structures.^[4]^

**
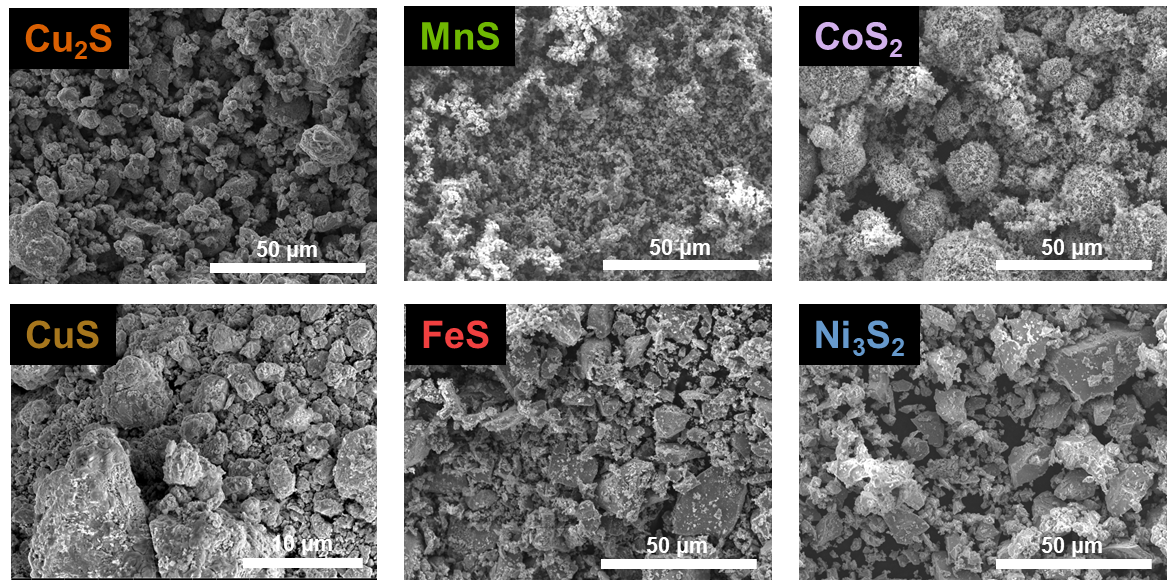
**

**Figure S1.** Scanning electron microscopy (SEM) images of bulk transition metal sulfide (TMS) powders.


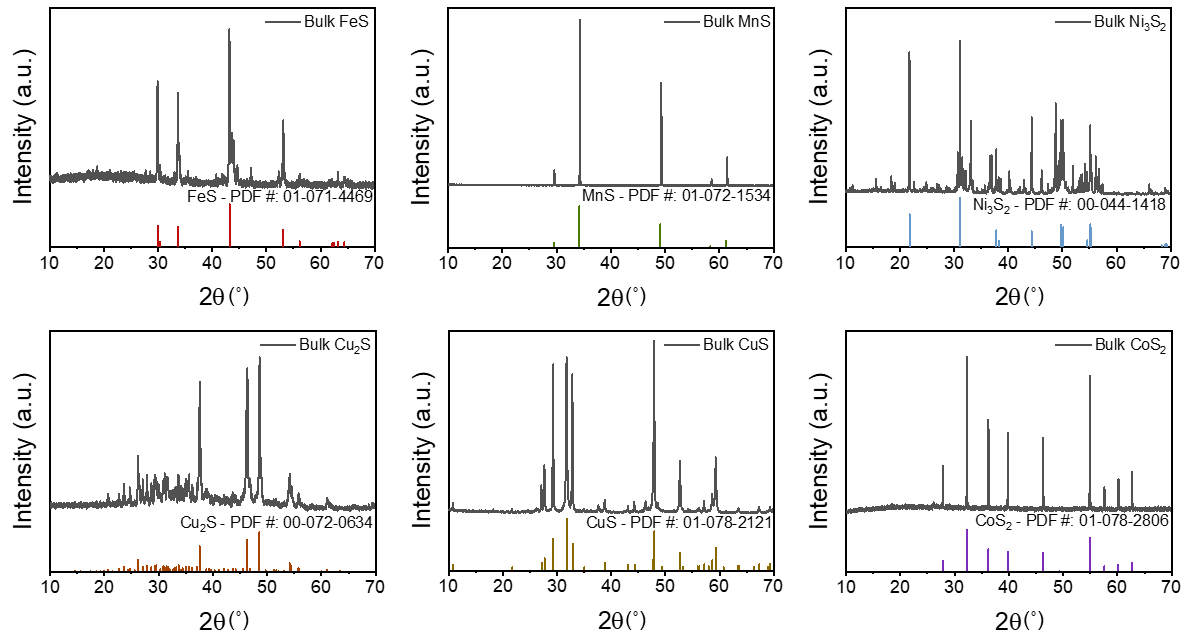


Figure S2. X-ray diffraction (XRD) patterns of bulk transition metal sulfide (TMS) powders. The distinguishable and sharp diffraction peaks of the bulk TMSs demonstrate their high crystallinity and intrinsic crystal structural characteristics. All crystal structure indexing, including lattice-plane assignments, was performed based on the PDF cards validated by the powder XRD results of the TMSs.

**
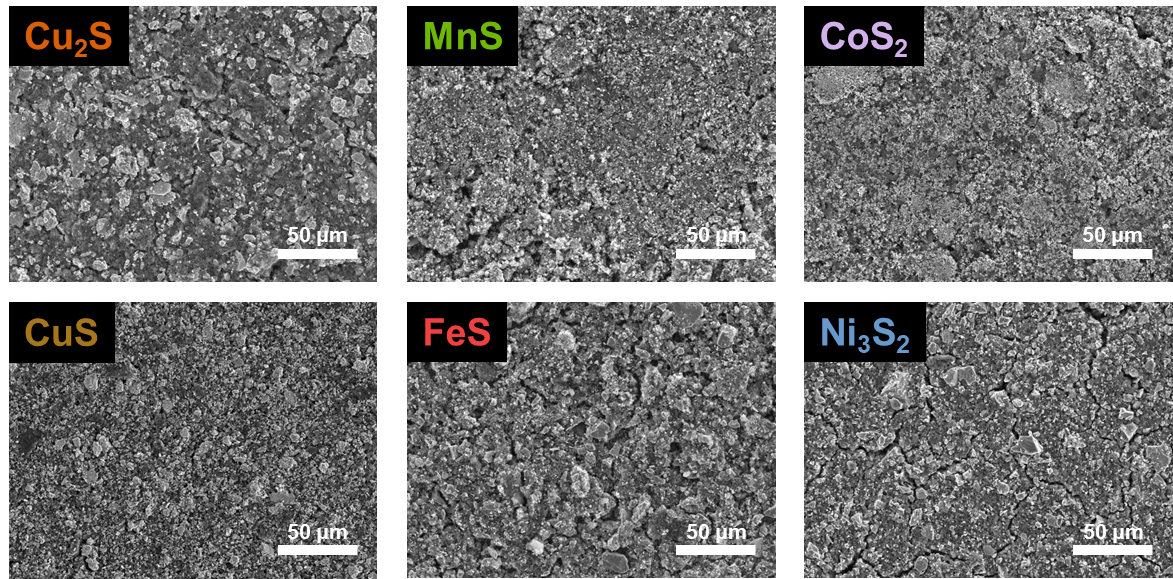
**

**Figure S3.** SEM images of the bulk TMS electrode surface.


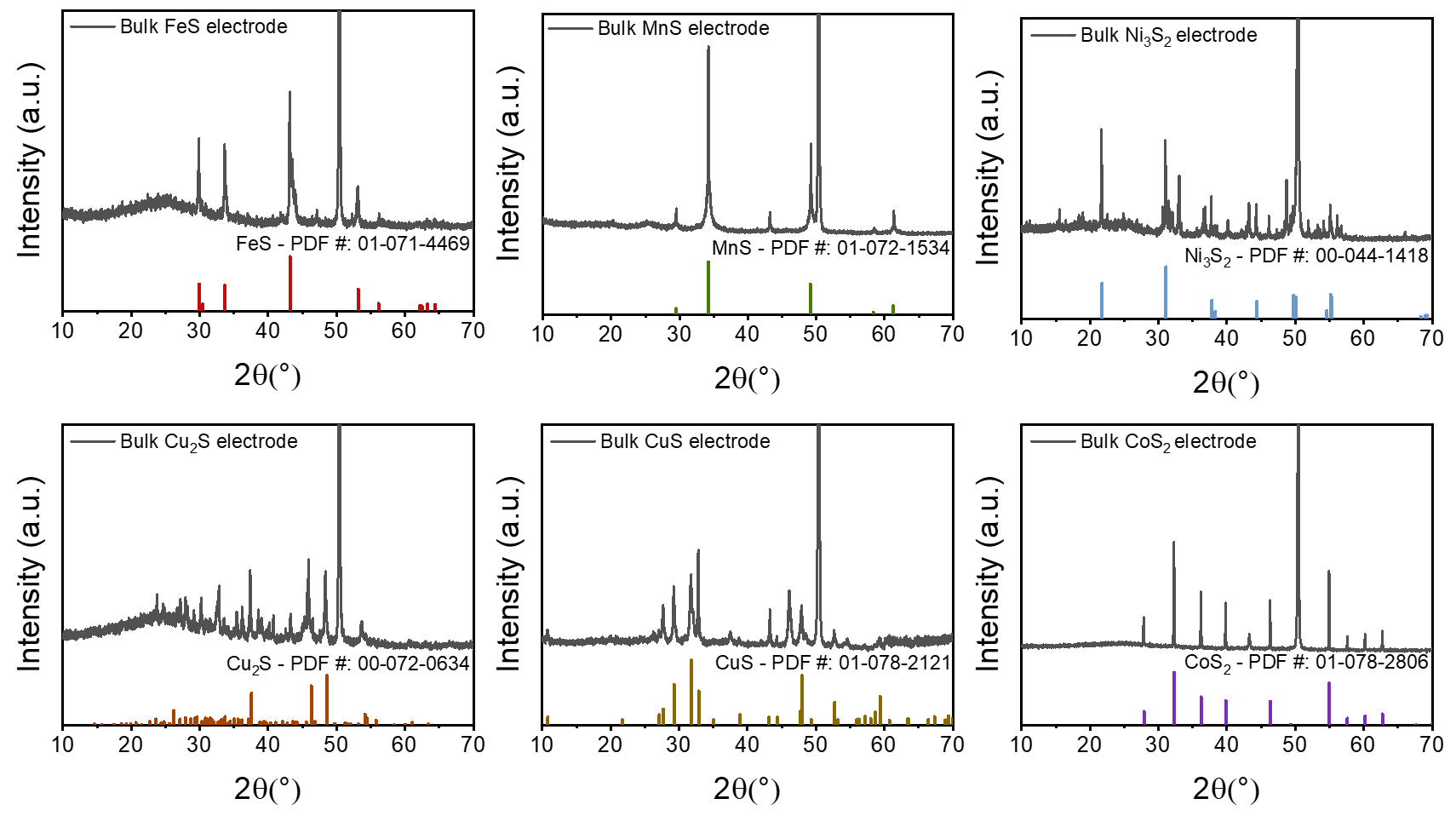


Figure S4. XRD patterns of bulk TMS anodes with copper current collectors (Cu-CC).


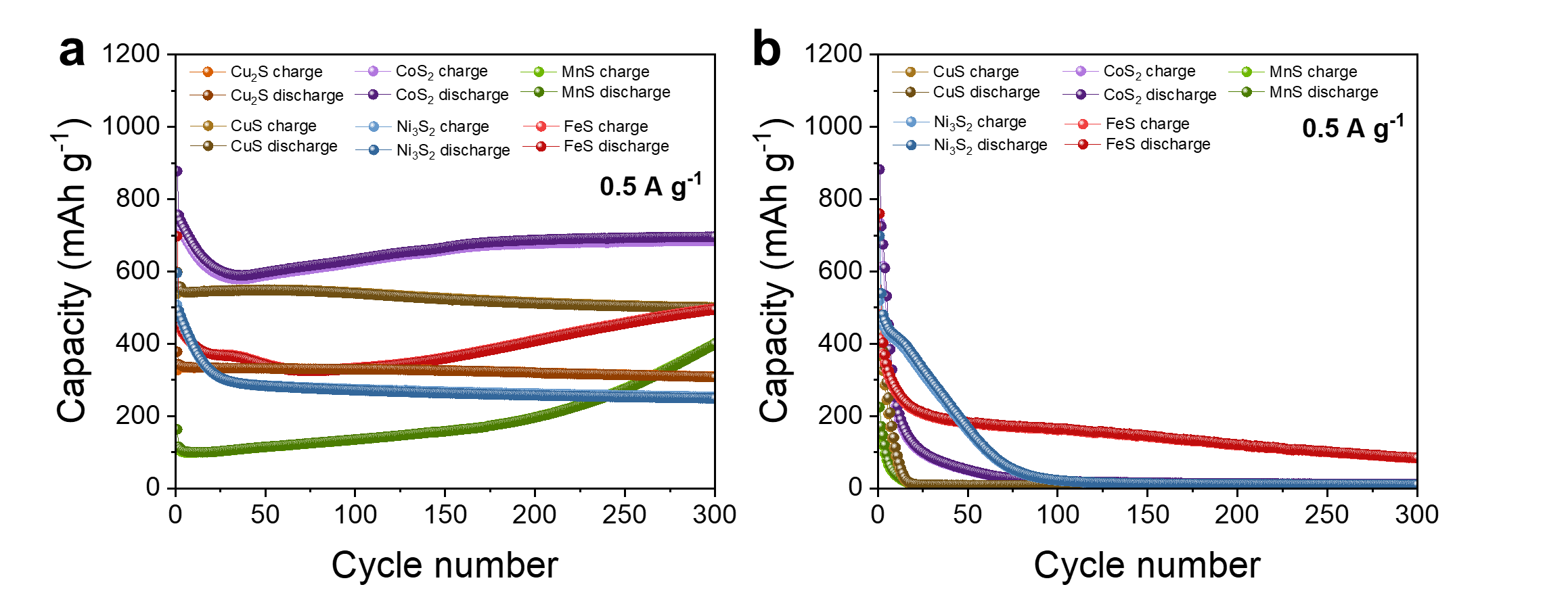


Figure S5. Electrochemical performance of bulk TMS electrodes with (a) ether-based electrolyte (1M NaPF_6_ in diglyme), and (b) carbonate-based electrolyte (1M NaPF_6_ in EC/DEC ,1:1 vol% + FEC 5wt%) in half-cell tests. All half-cell tests were conducted with the identical voltage window (0.05 V–2.6 V), and current density (0.5 A g^-1^).


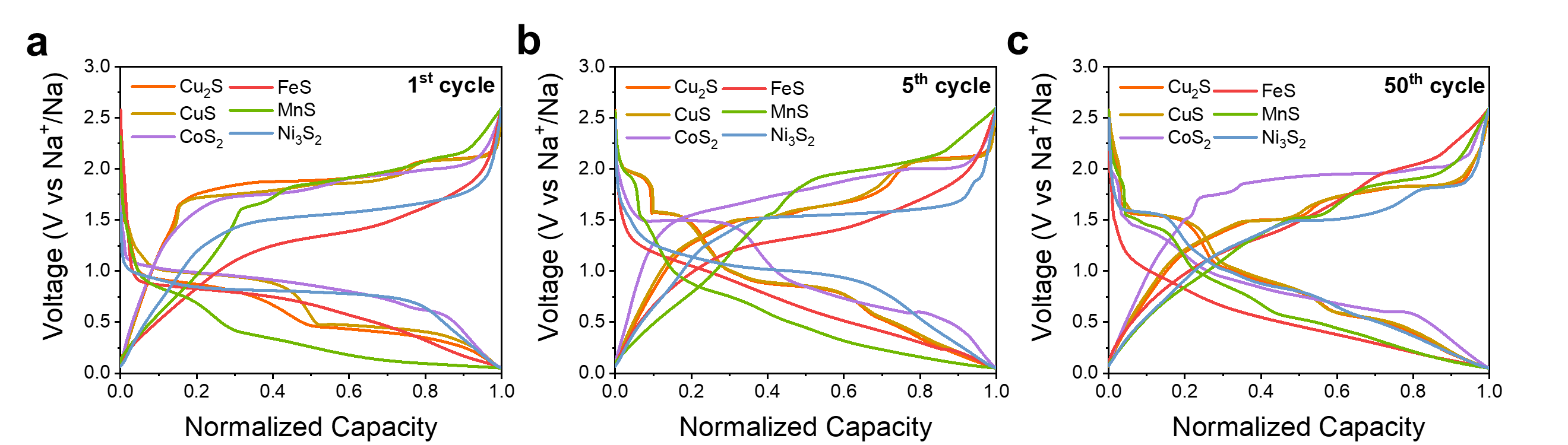


Figure S6. Normalized and merged charge–discharge profiles of TMS anodes at (a) 1^st^ cycle, (b) 5^th^ cycle, and (c) 50^th^ cycle. It has been observed that, during the initial cycles of charge-discharge, each TMS anode exhibits a distinct intrinsic charge-discharge profile. However, it is noteworthy that these profiles tend to converge towards a more comparable characteristic as they continue through subsequent cycles.


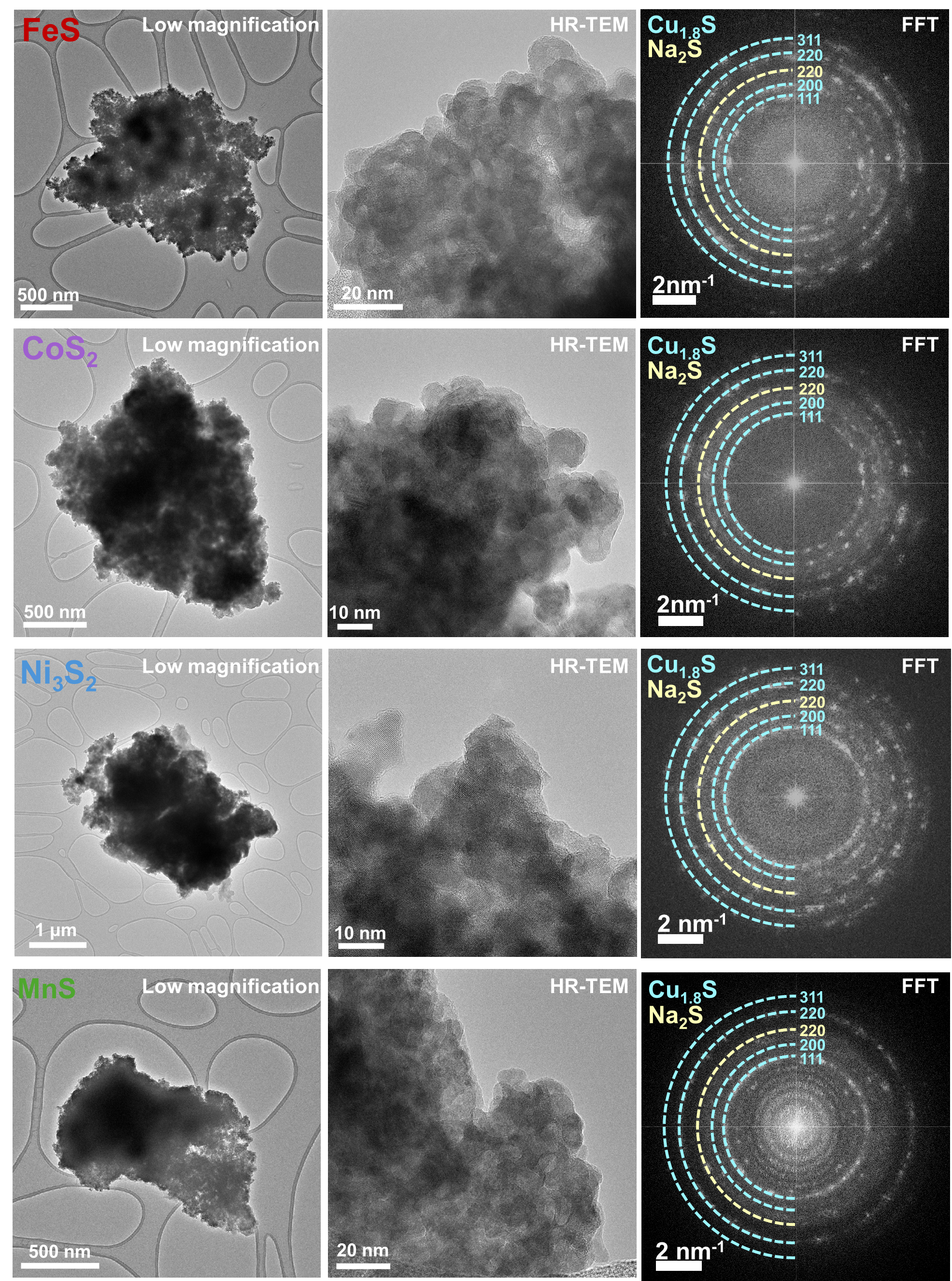


Figure S7. Low-magnification TEM images (left), high-resolution TEM (HR-TEM) images (middle), and corresponding fast Fourier transform (FFT) patterns (right) of bulk TMS anodes after 300 cycles. The ring patterns in the FFT of the HR-TEM images indicate their polycrystalline nature with nano-sized grains.

**
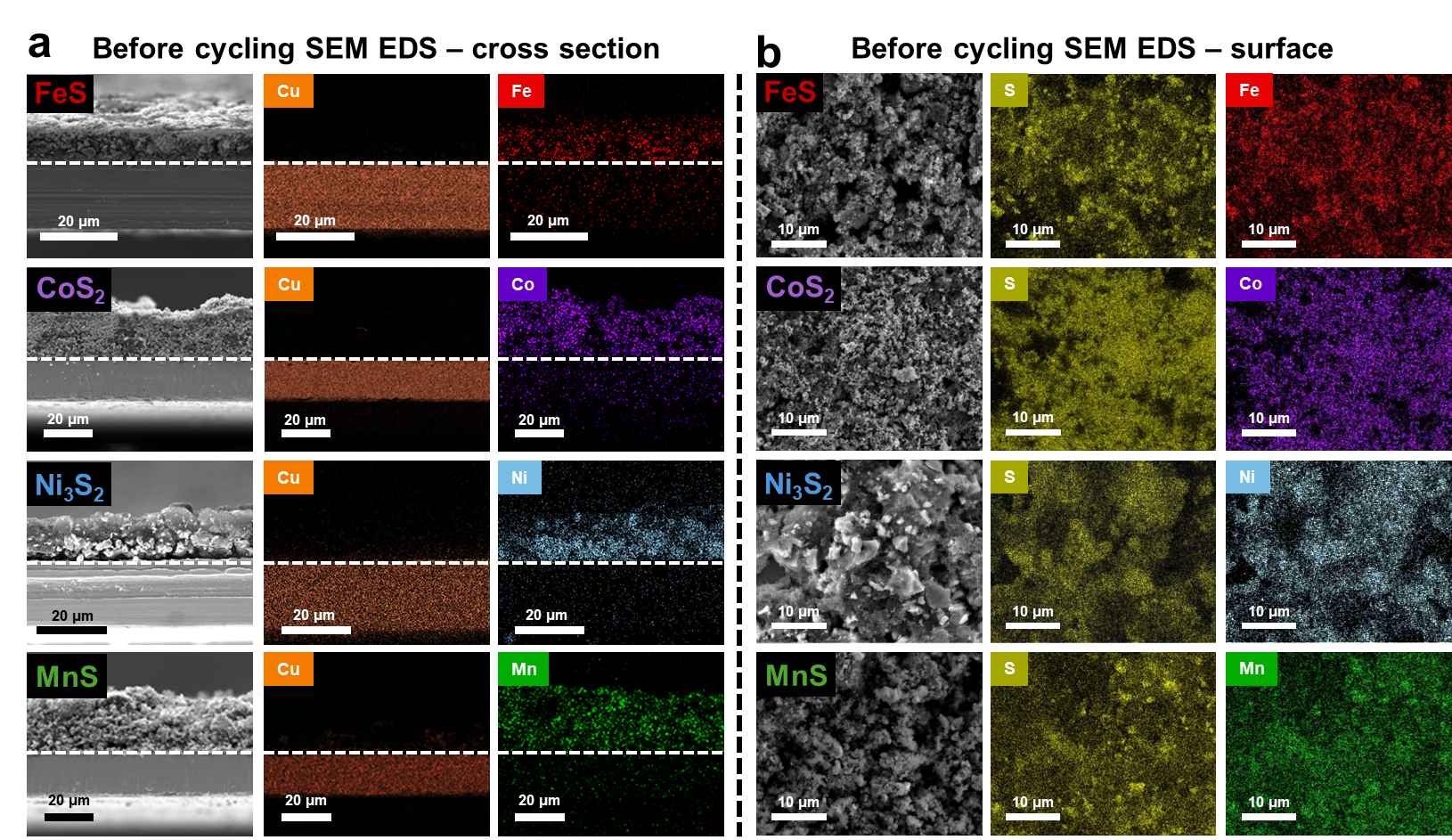
**

Figure S8. (a) Cross sectional SEM-EDS, and (b) surface SEM-EDS elemental mapping results of bulk TMS anodes before charge–discharge cycles.


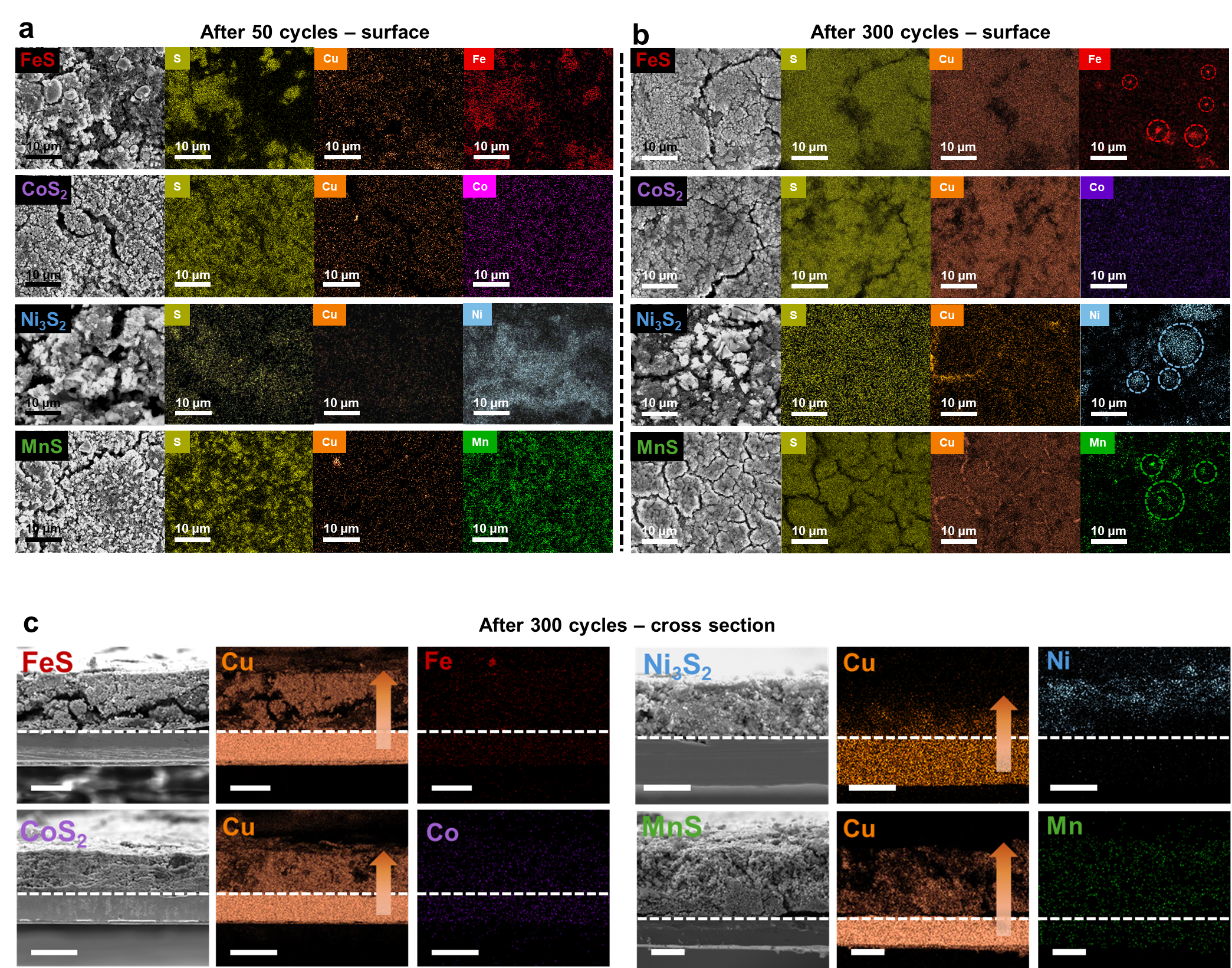


Figure S9. Surface SEM-EDS elemental mapping results of bulk TMS anodes after (a) 50 cycles, and (b) 300 cycles. (c) Cross-sectional SEM-EDS elemental mapping results of bulk TMS anodes after 300 cycles (scale bar, 20 µm). The Cu element was not readily detectable on the electrode surface after 50 cycles, but became the dominant element detected on the electrode surface after 300 cycles. For FeS, Ni_3_S_2_, and MnS electrodes after 300 cycles, the original TM elements are detectable in localized regions (highlighted with dashed circles in Figure S9b).


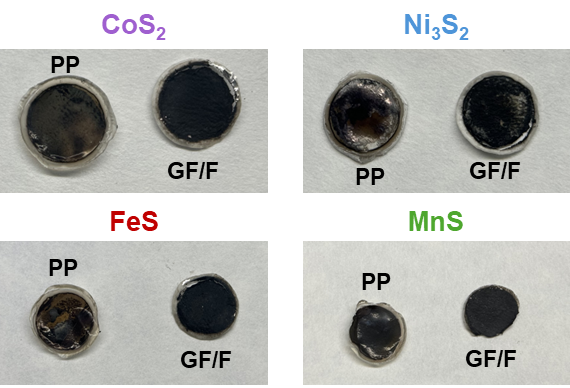


Figure S10. Optical photograph images of separators from bulk TMS anodes after 300 cycles (PP: Celgard 2400)


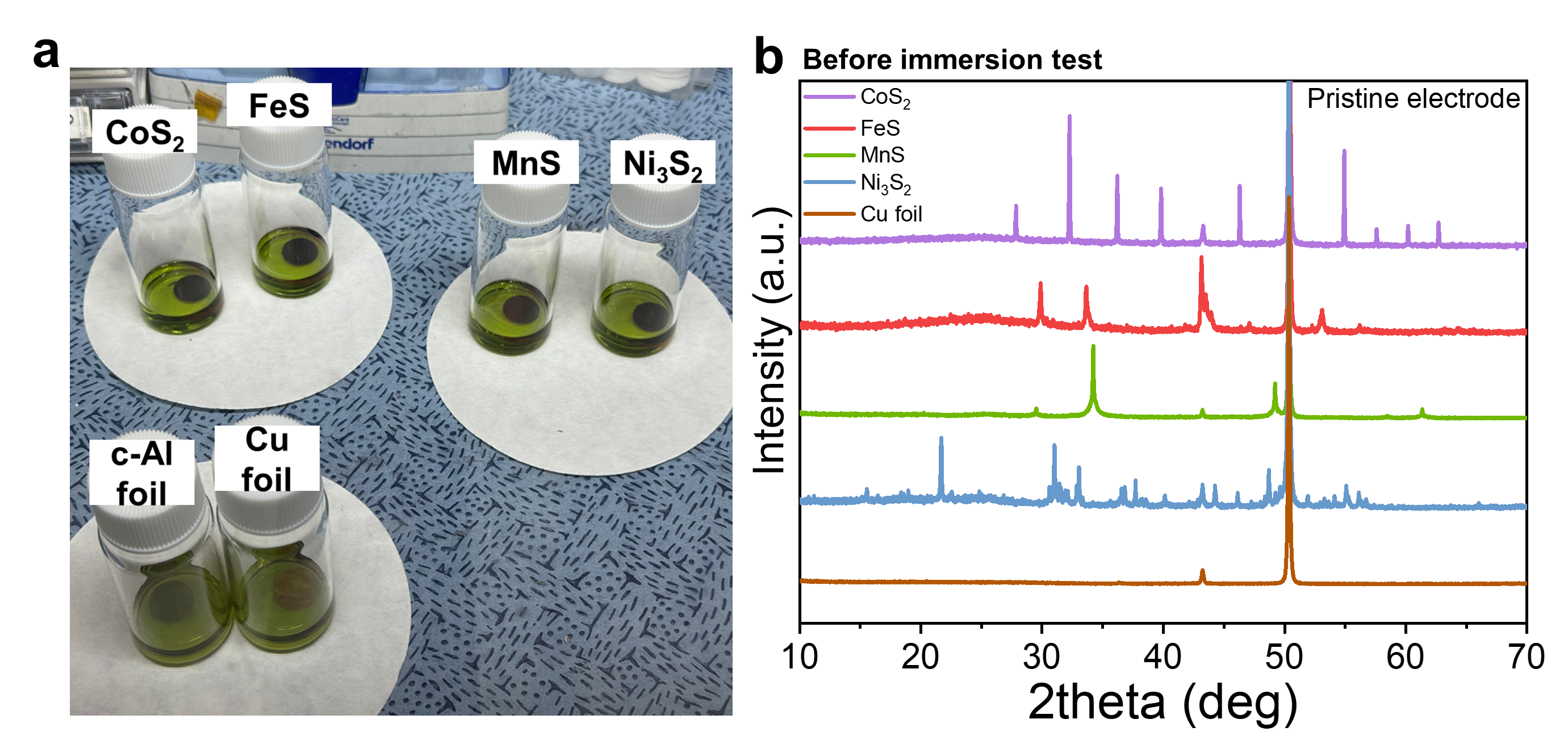


Figure S11. (a) Optical photographs of the solution immediately after initiating the immersion test of the TMS electrodes. (b) Ex-situ XRD results of pristine bulk TMS electrodes, and Cu foil.


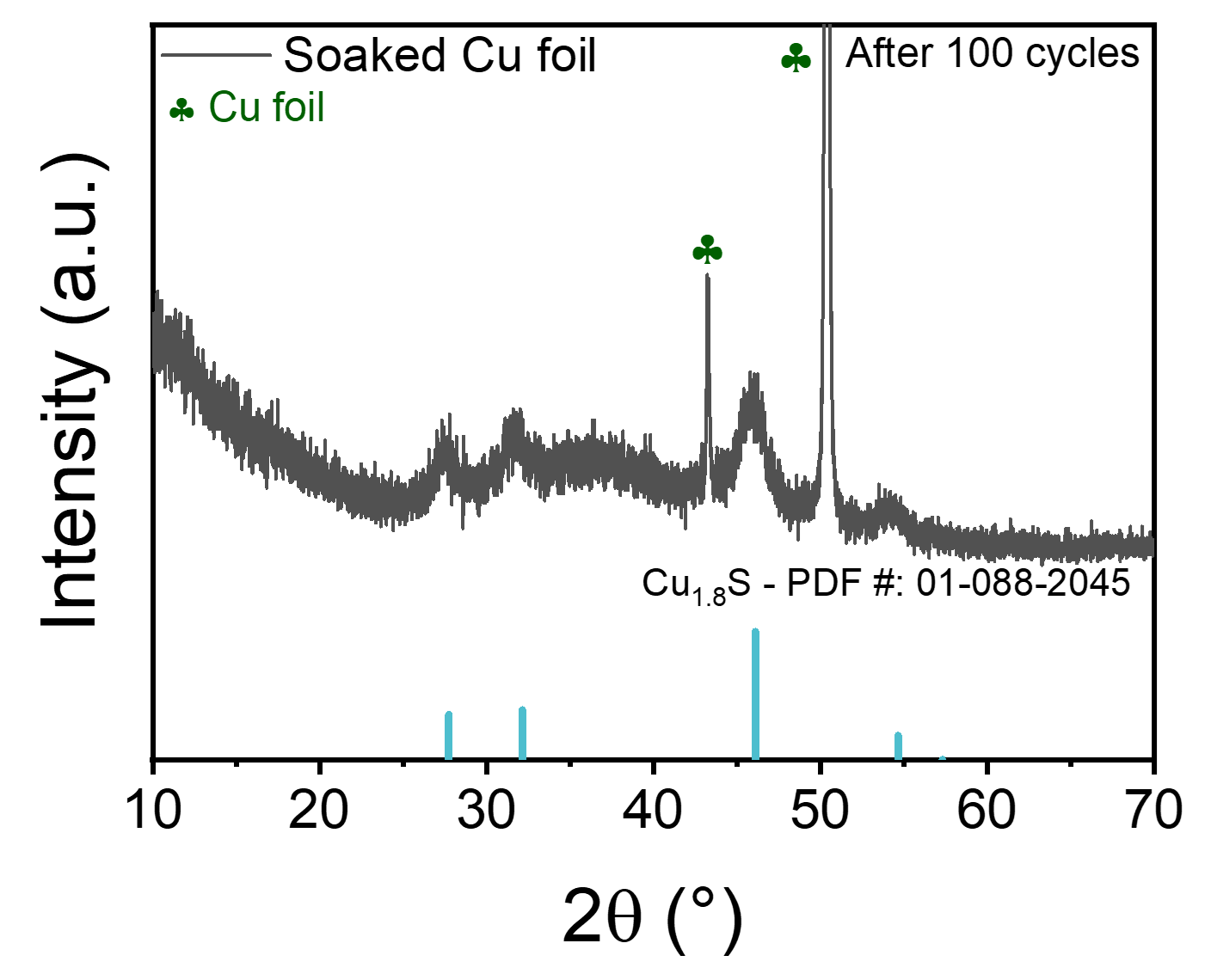


Figure S12. Ex-situ XRD result of reaction product-coated Cu foil after 100 cycles. The initial NaCu_5_S_3_ and Cu_2_S phases undergo phase transition to Cu_1.8_S during charge-discharge cycles, which is analogous to the other TMS anodes with the Cu-CC system.


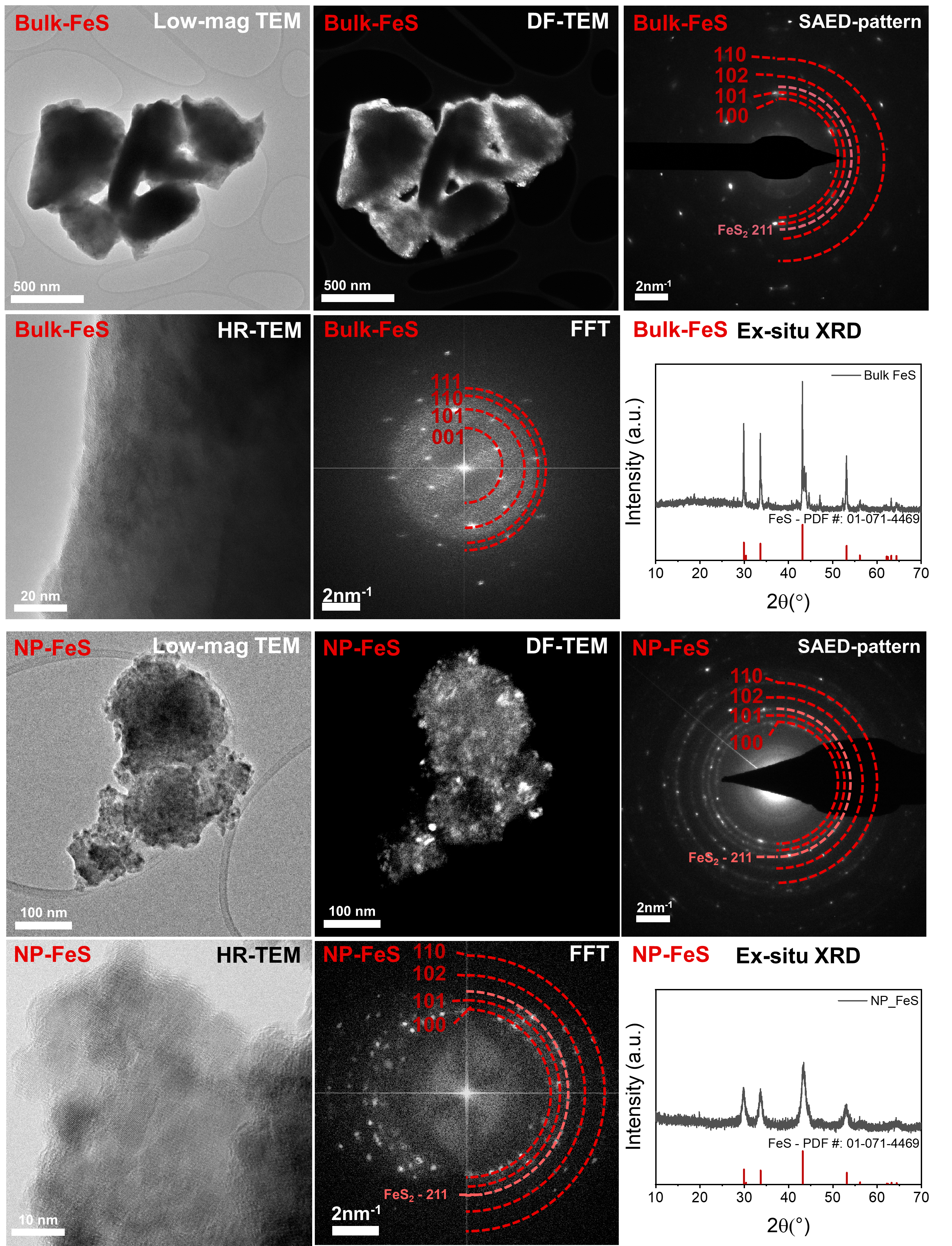


Figure S13. Crystal structure and morphological characterization of bulk-FeS and NP-FeS. Compared with bulk-FeS, NP-FeS exhibits ring-like SAED patterns and nano-sized grains observed by HR-TEM, which is further supported by the corresponding FFT pattern. The multiple diffracted spots forming ring-like patterns in the FFT of the HR-TEM and in the SAED images indicate the nano-sized polycrystalline nature of the NP-FeS.


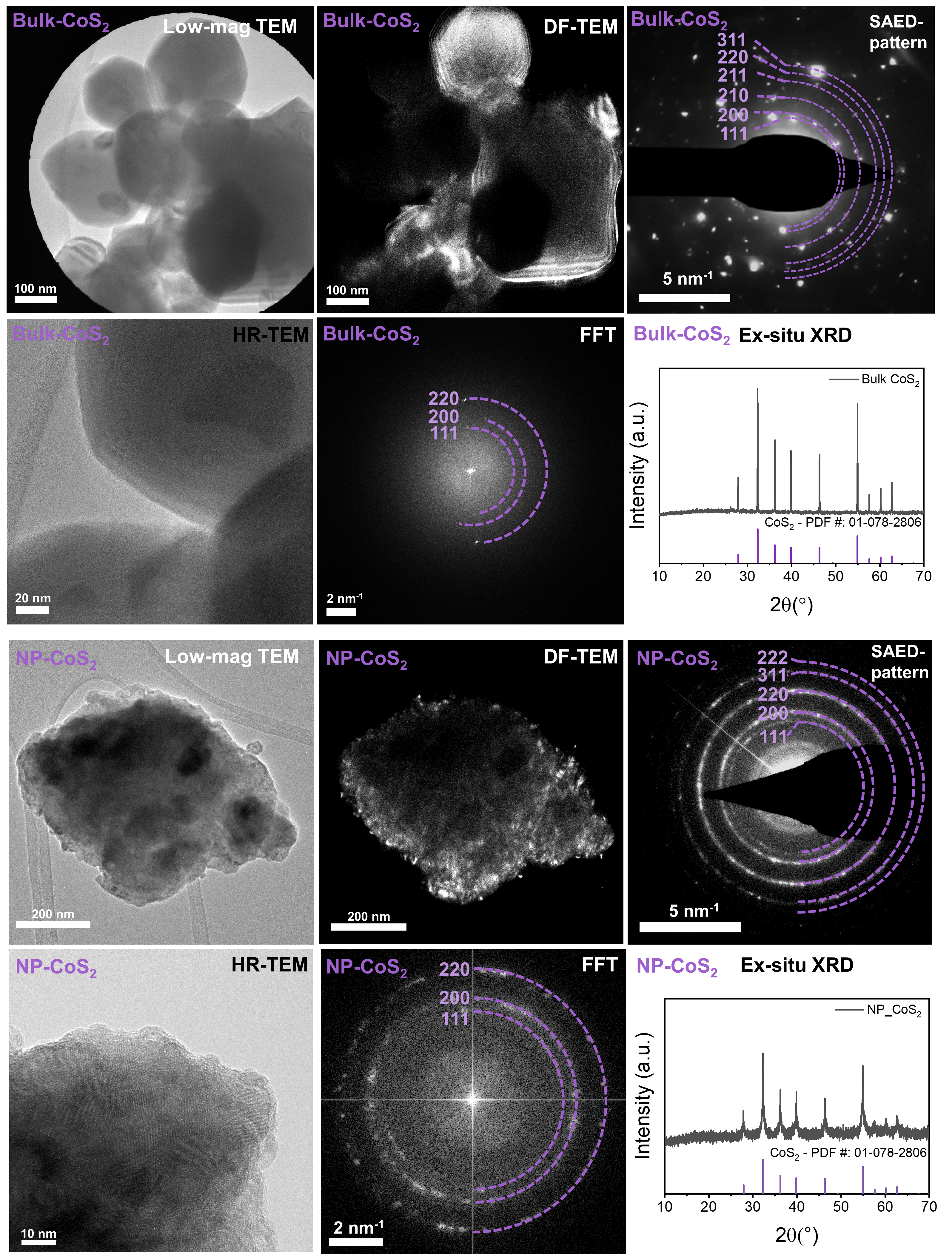


Figure S14. Crystal structure and morphological characterization of bulk-CoS_2_ and NP-CoS_2_. Compared with bulk-CoS_2_, NP-CoS_2_ exhibits ring-like SAED patterns and nano-sized grains observed by HR-TEM, which is further supported by the corresponding FFT pattern. The multiple diffracted spots forming ring-like patterns in the FFT of the HR-TEM and in the SAED images indicate the nano-sized polycrystalline nature of the NP-CoS_2_. Similar to the FeS case, the increase in the FWHM of the NP-CoS_2_ XRD peak indicates a reduction in grain size, consistent with the Scherrer equation.


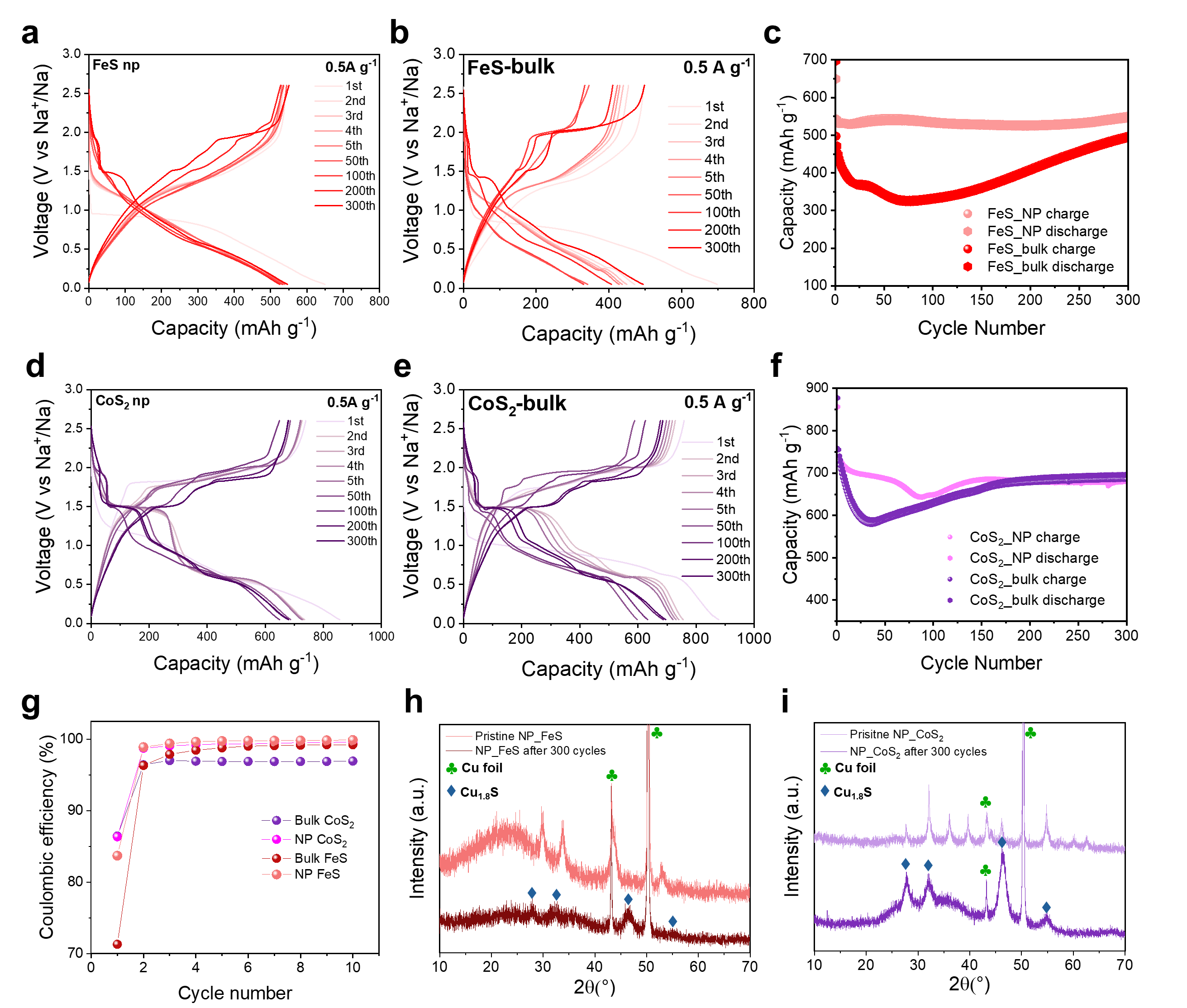


Figure S15. Charge-discharge profiles of (a) NP-FeS and (b) bulk-FeS. (c) Cyclic performance of NP-FeS and bulk-FeS with Cu-CC. Charge-discharge profiles of (d) NP-CoS_2_ and (e) bulk-CoS_2_. (c) Cyclic performance of NP-CoS_2_ and bulk-CoS_2_ with Cu-CC. (g) Coulombic efficiency of NP-FeS, bulk-FeS, NP-CoS_2_, and bulk-CoS_2_. (h) Ex-situ XRD patterns of the pristine NP-FeS electrode and after 300 cycles. (i) Ex-situ XRD patterns of the pristine NP-CoS_2_ electrode and after 300 cycles. Regardless of the use of nanoparticle strategies, a phase transition to Cu_1.8_S ultimately occurred after 300 cycles.


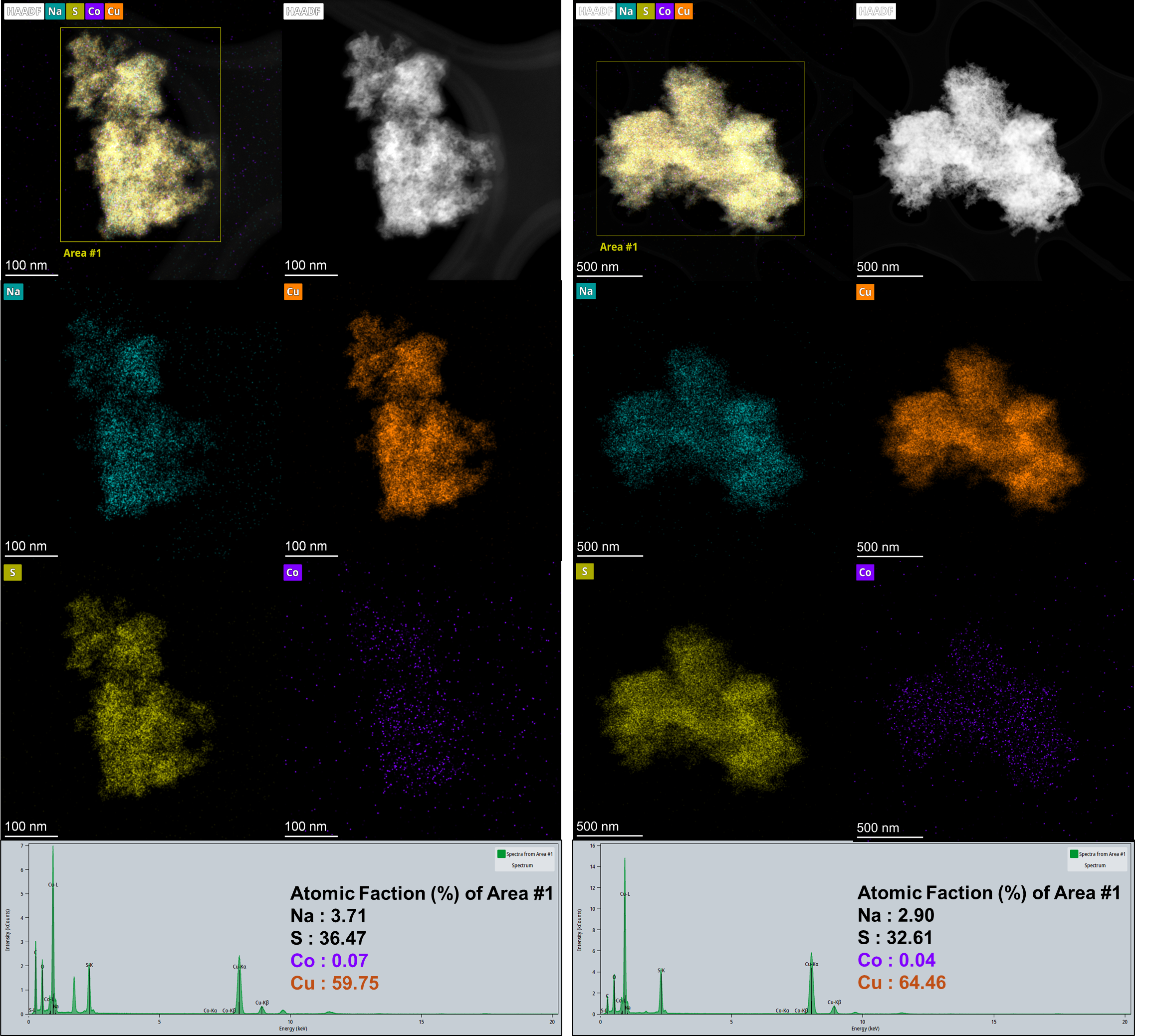


**Figure S16.** STEM-EDS results of bulk-CoS_2_ electrode with Cu-CC after 300 cycles.


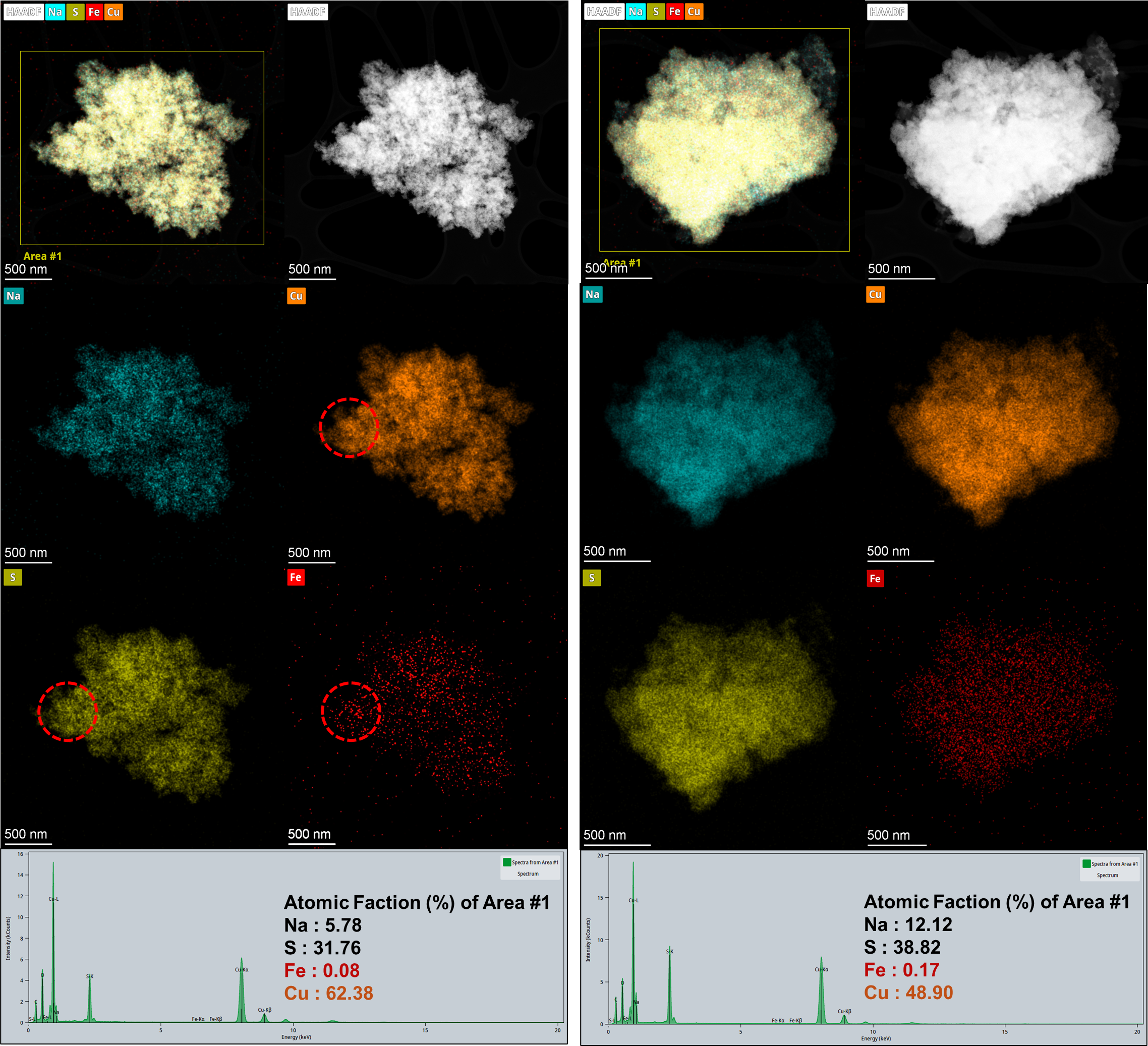


**Figure S17.** STEM-EDS results of bulk-FeS electrode with Cu-CC after 300 cycles. The red dashed line indicates the isolated Fe element.

**
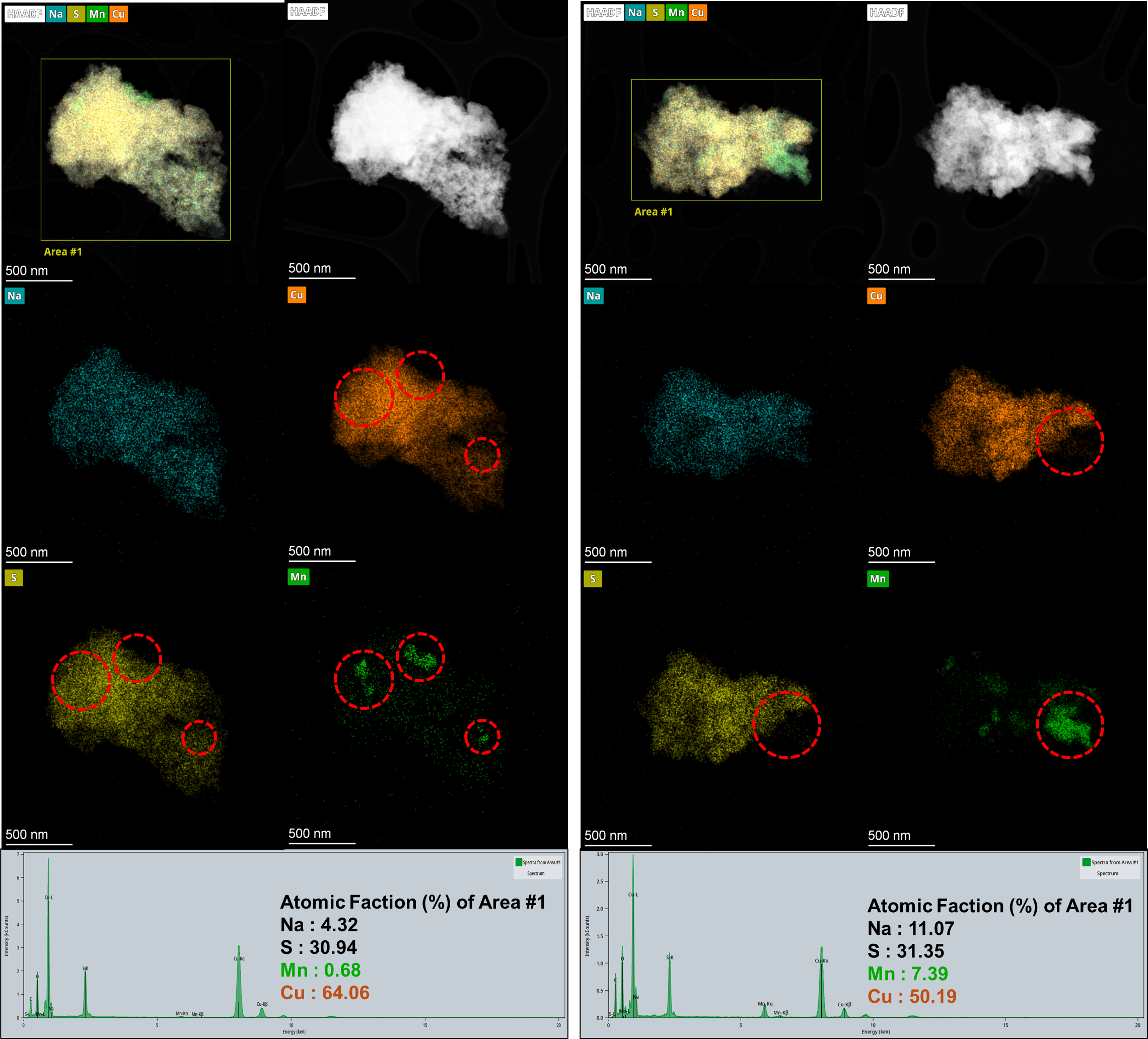
**

**Figure S18.** STEM-EDS results of bulk-MnS electrode with Cu-CC after 300 cycles. The red dashed line indicates the isolated Mn element.

**
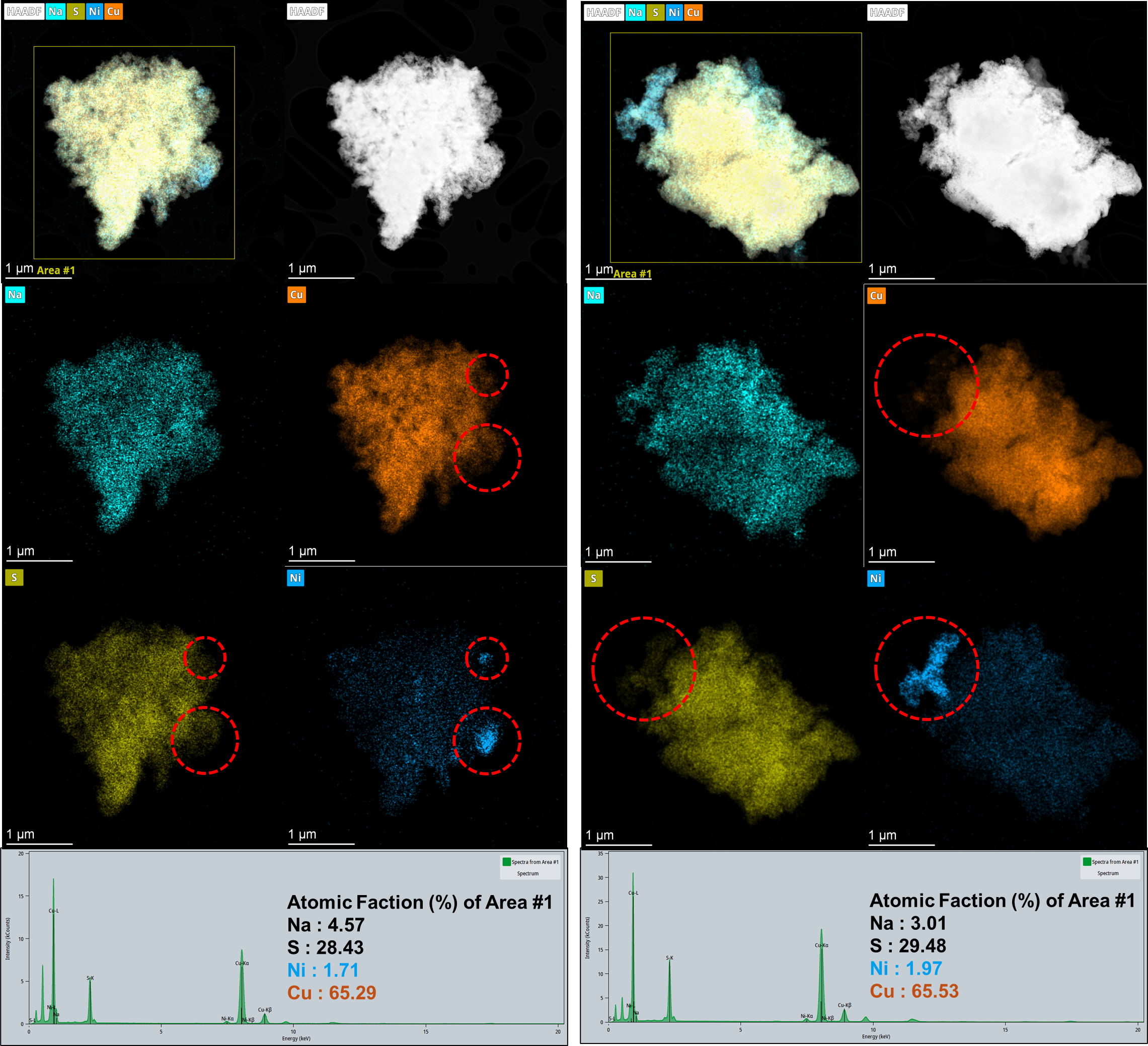
**

**Figure S19.** STEM-EDS results of bulk-Ni_3_S_2_ electrode with Cu-CC after 300 cycles. The red dashed line indicates the isolated Ni element.

**
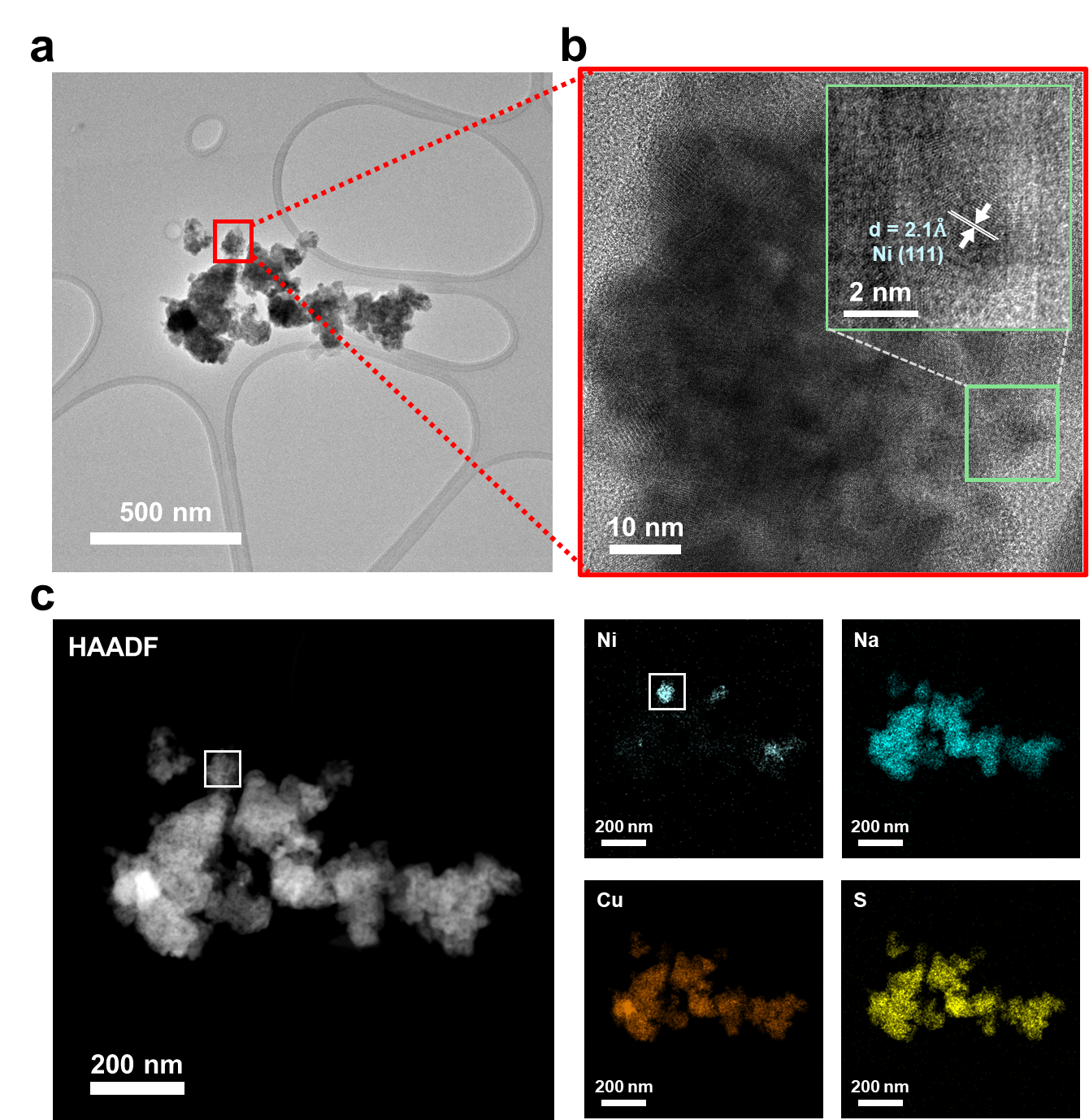
**

**Figure S20. (a-b)** Low-magnification TEM and HR-TEM images of Ni_3_S_2_ electrode after 300 cycles. **(c)** STEM-EDS mapping images of Ni_3_S_2_ after 300 cycles. HR-TEM images were obtained from the region where the Ni element was isolated, as confirmed by the STEM-EDS mapping results. The measured interlayer distance was 0.21 nm, which corresponds to the (111) plane of Ni.

**
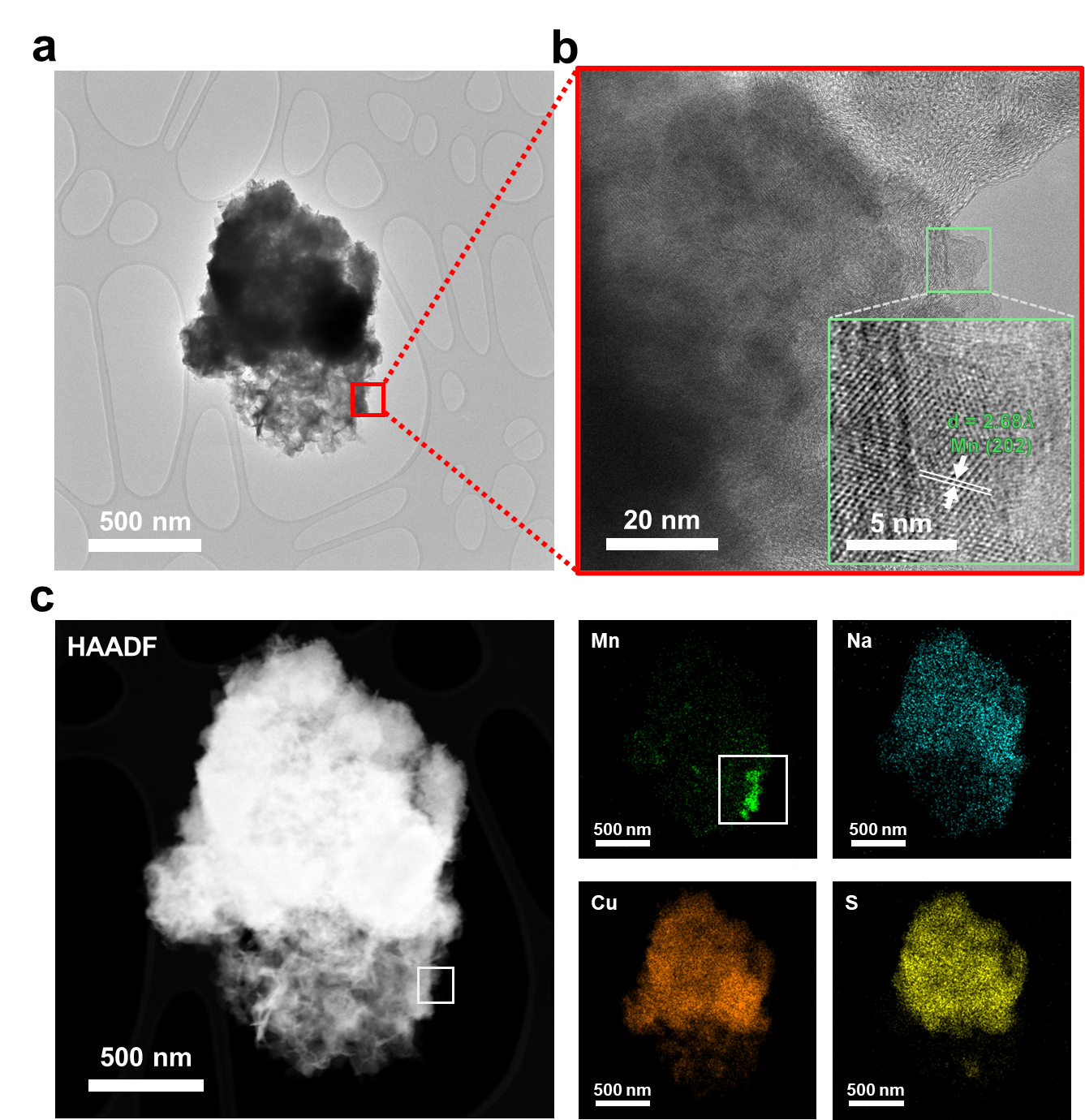
**

**Figure S21. (a-b)** Low-magnification TEM and HR-TEM images of MnS electrode after 300 cycles. **(c)** STEM-EDS mapping images of MnS after 300 cycles. HR-TEM images were obtained from the region where the Mn element was isolated, as confirmed by the STEM-EDS mapping results. The measured interlayer distance was 0.268 nm, which corresponds to the (202) plane of Mn.

**
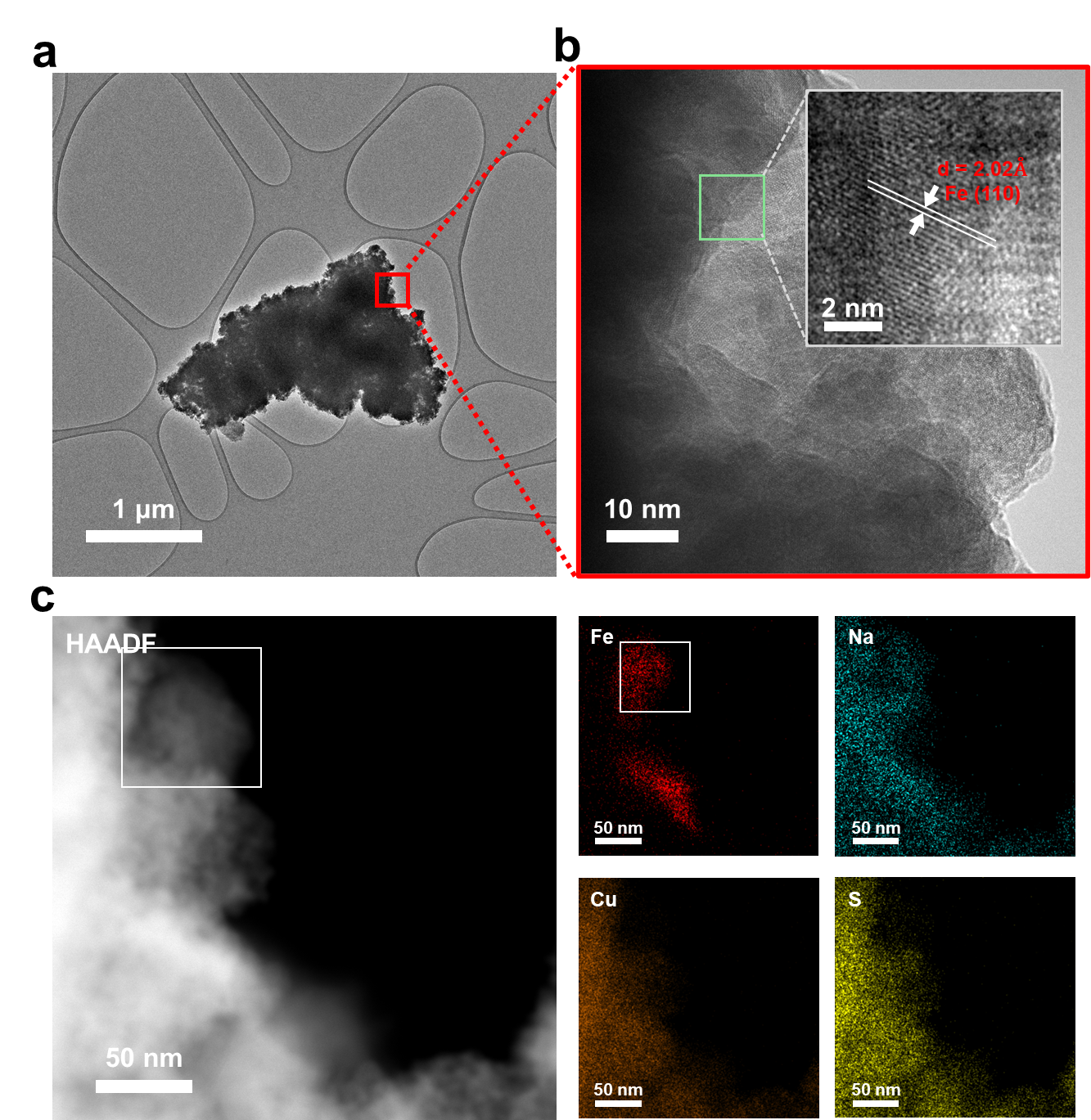
**

**Figure S22. (a-b)** Low-magnification TEM and HR-TEM images of FeS electrode after 300 cycles. **(c)** STEM-EDS mapping images of FeS after 300 cycles. HR-TEM images were obtained from the region where the Fe element was isolated, as confirmed by the STEM-EDS mapping results. The measured interlayer distance was 0.202 nm, which corresponds to the (110) plane of Fe.

**
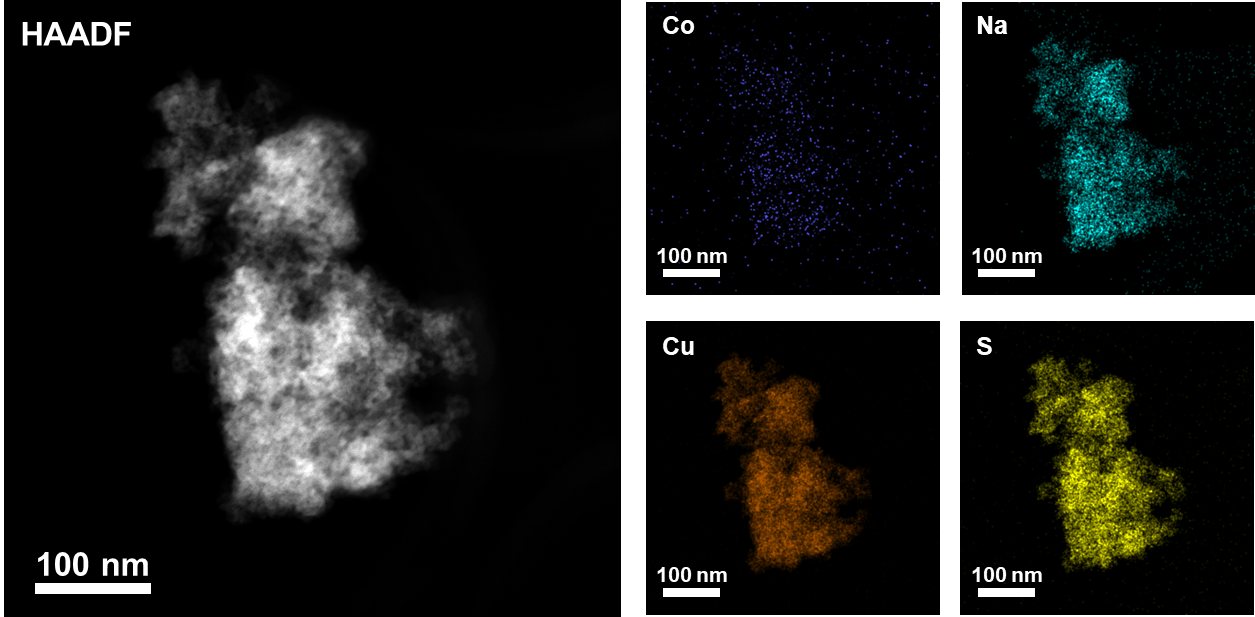
**

**Figure S23.** STEM EDS elemental mapping images of CoS_2_ after 300 cycles. The residual Co content was insufficient to resolve any isolated Co-rich regions.

**
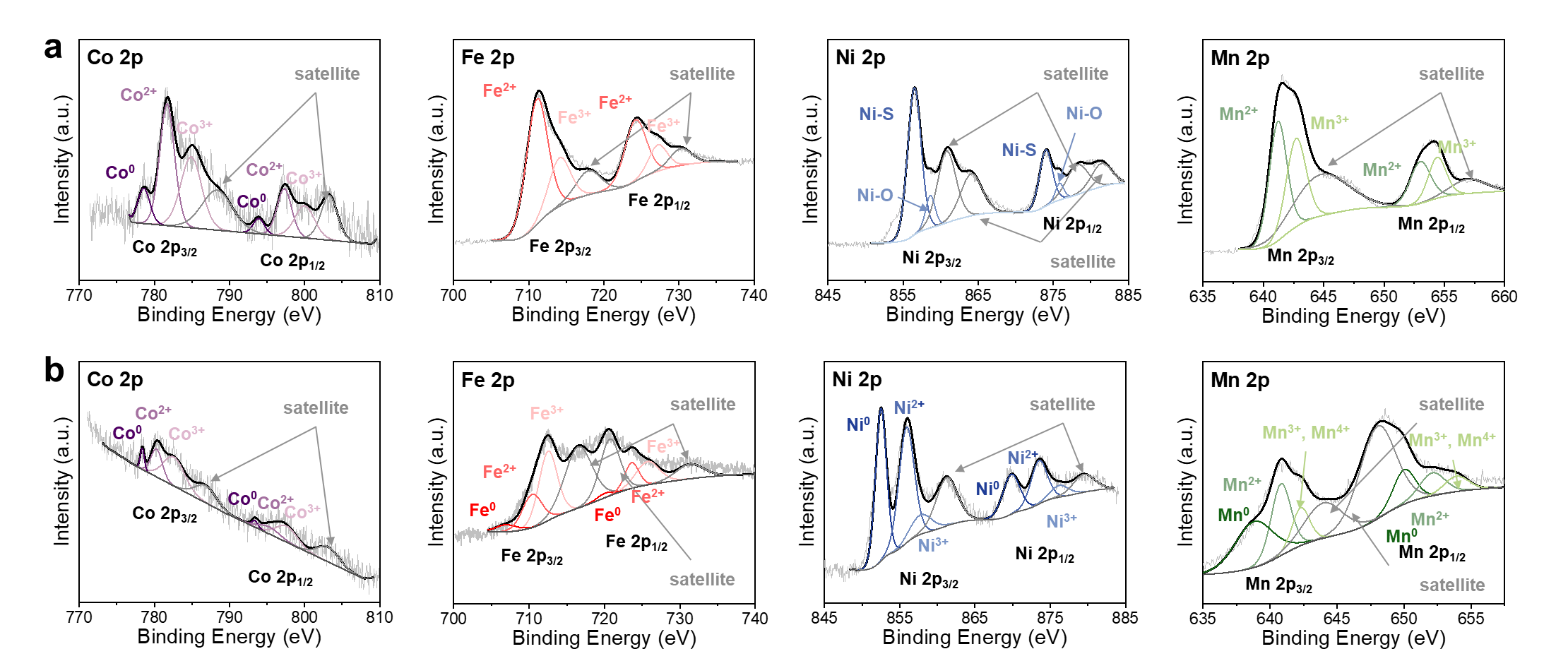
**

**Figure S24.** XPS spectra of CoS_2_, FeS, Ni_3_S_2_, and MnS electrodes (a) before and (b) after 300 cycles.

**
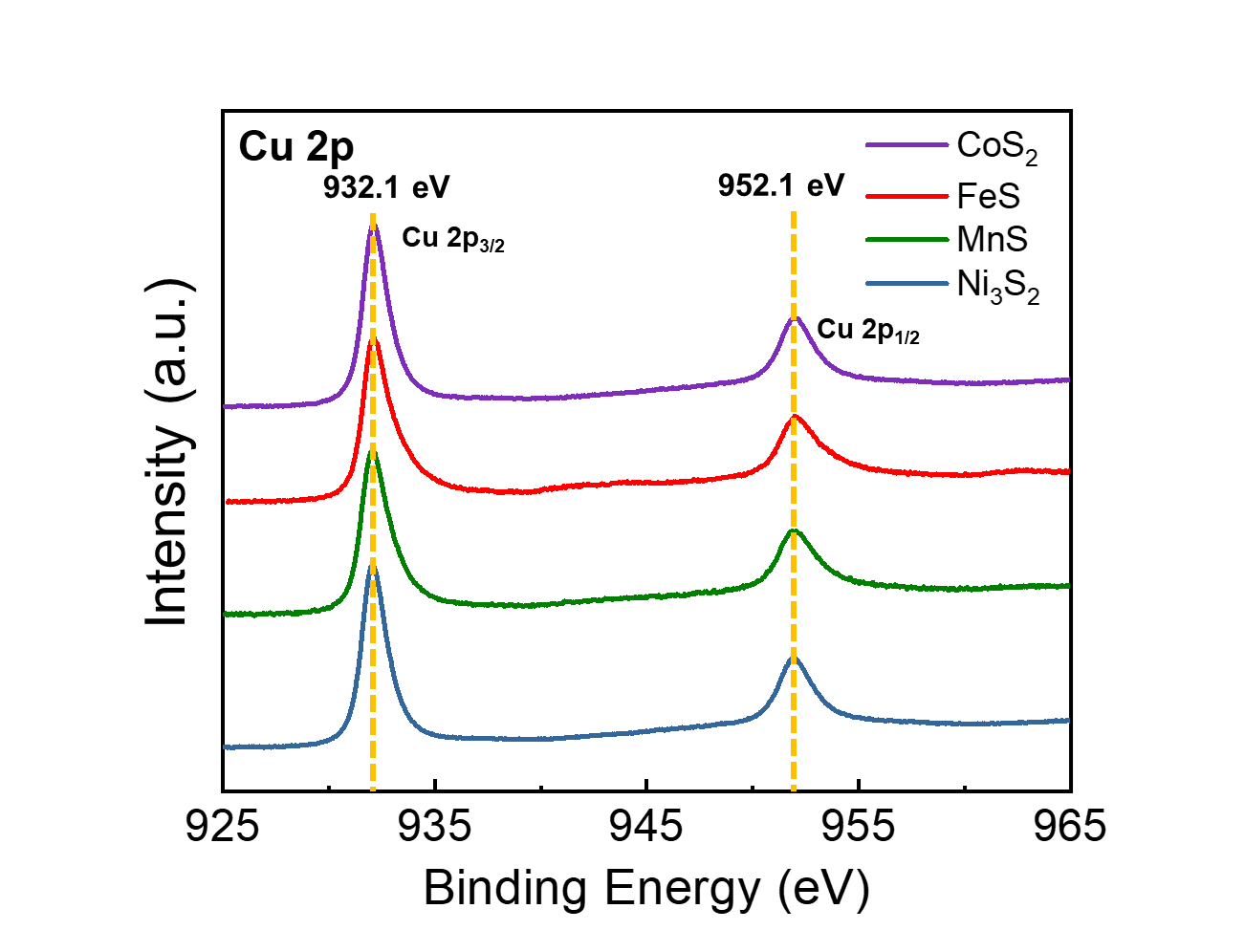
**

**Figure S25.** Cu 2p XPS dataset of TMS electrodes after 300 cycles. After 300 cycles, the Cu binding energies for all TMS electrodes were identical and consistent with the reported binding energy of Cu_1.8_S.^[5,6]^

**
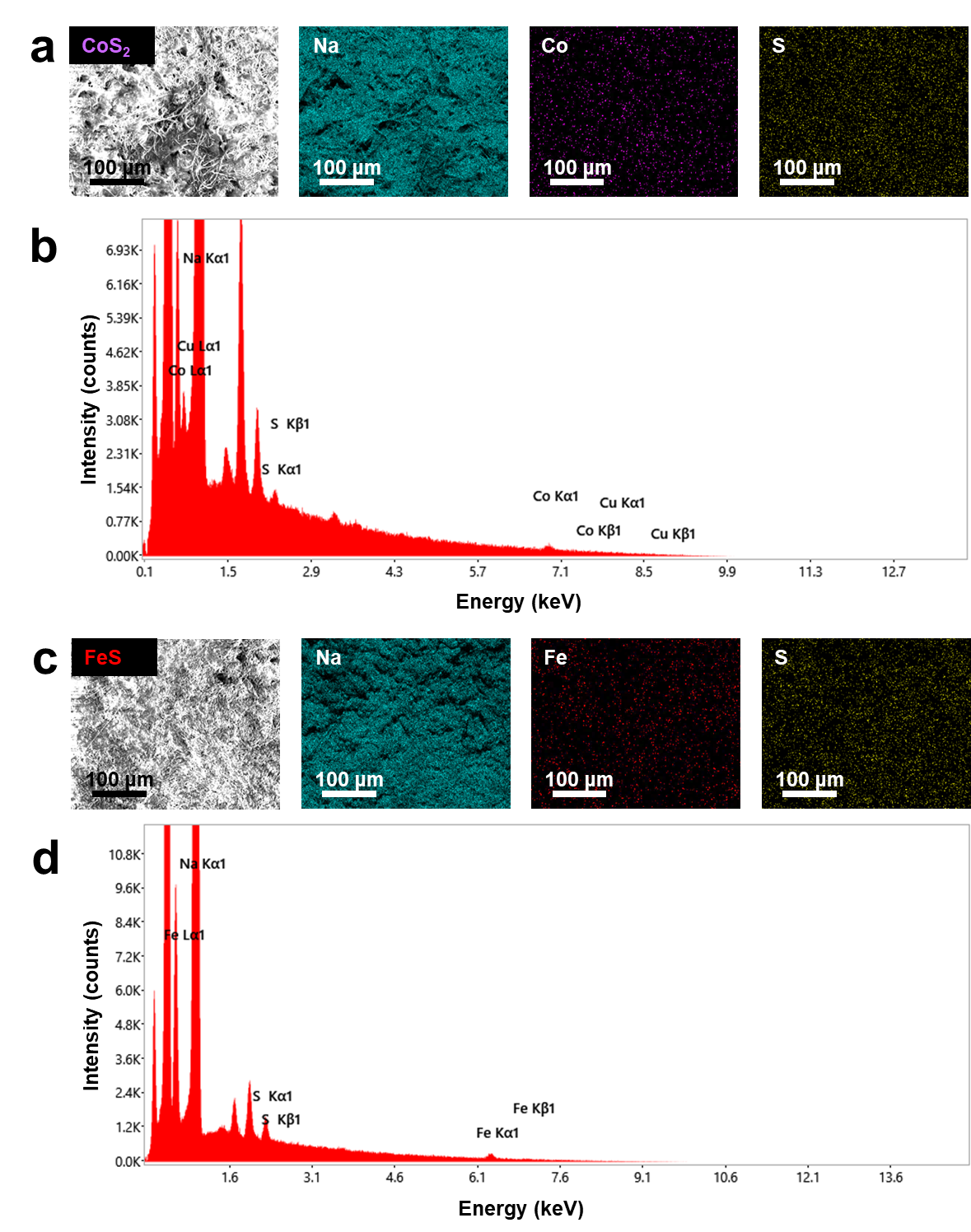
**

**Figure S26. (a, b)** SEM-EDS mapping results and the corresponding EDS spectra of the counter electrode (Na metal) from the bulk-CoS_2_ cell after 300 cycles. **(c, d)** SEM-EDS mapping results and the corresponding EDS spectra of the counter electrode from the bulk-FeS cell after 300 cycles.

**
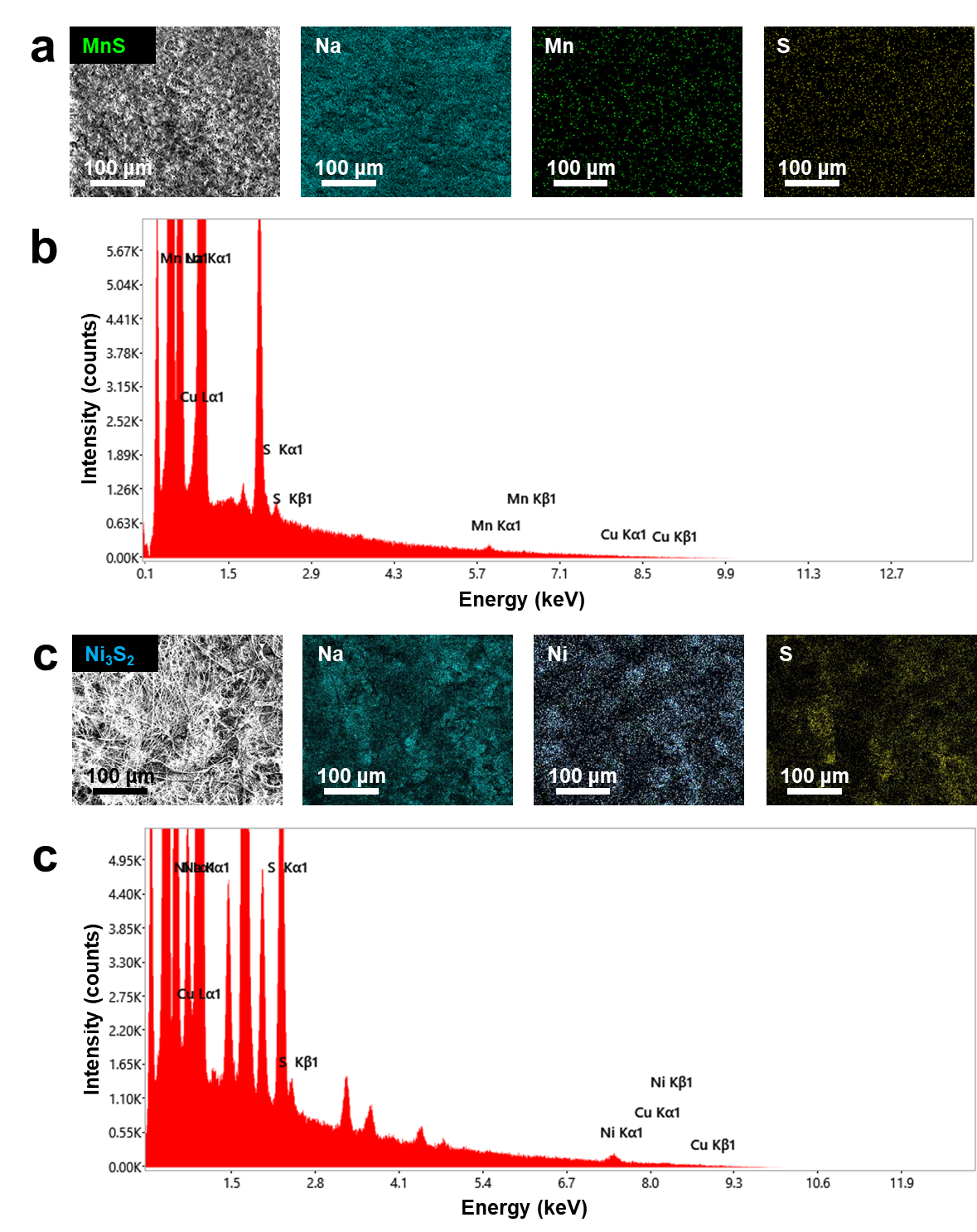
**

**Figure S27. (a, b)** SEM-EDS mapping results and the corresponding EDS spectra of the counter electrode (Na metal) from the bulk-MnS cell after 300 cycles. **(c, d)** SEM-EDS mapping results and the corresponding EDS spectra of the counter electrode from the bulk-Ni_3_S_2_ cell after 300 cycles.

**
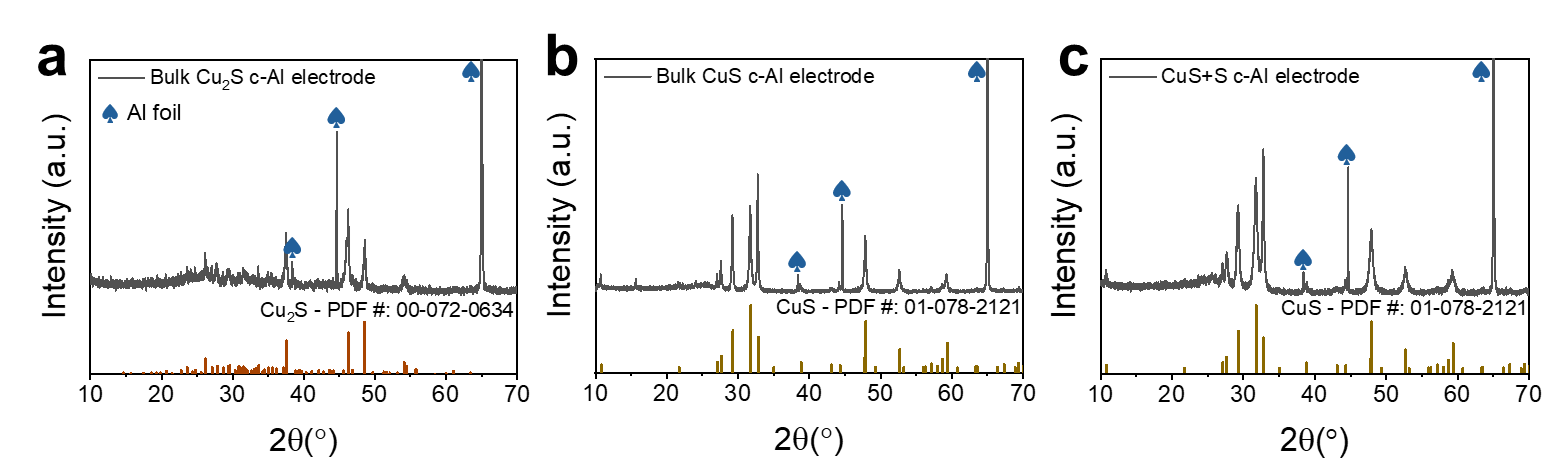
**

**Figure S28.** Ex**-**situ XRD patterns of pristine **(a)** bulk Cu_2_S, **(b)** bulk CuS, and **(c)** CuS+S electrode supported on a carbon-coated-Aluminum current collector (c-Al-CC). The CuS+S electrode was prepared by ball-milling CuS and S in a 1:1 molar ratio. As confirmed by the ex-situ XRD analysis, no change in the crystal structure of the mixed CuS was observed.

**
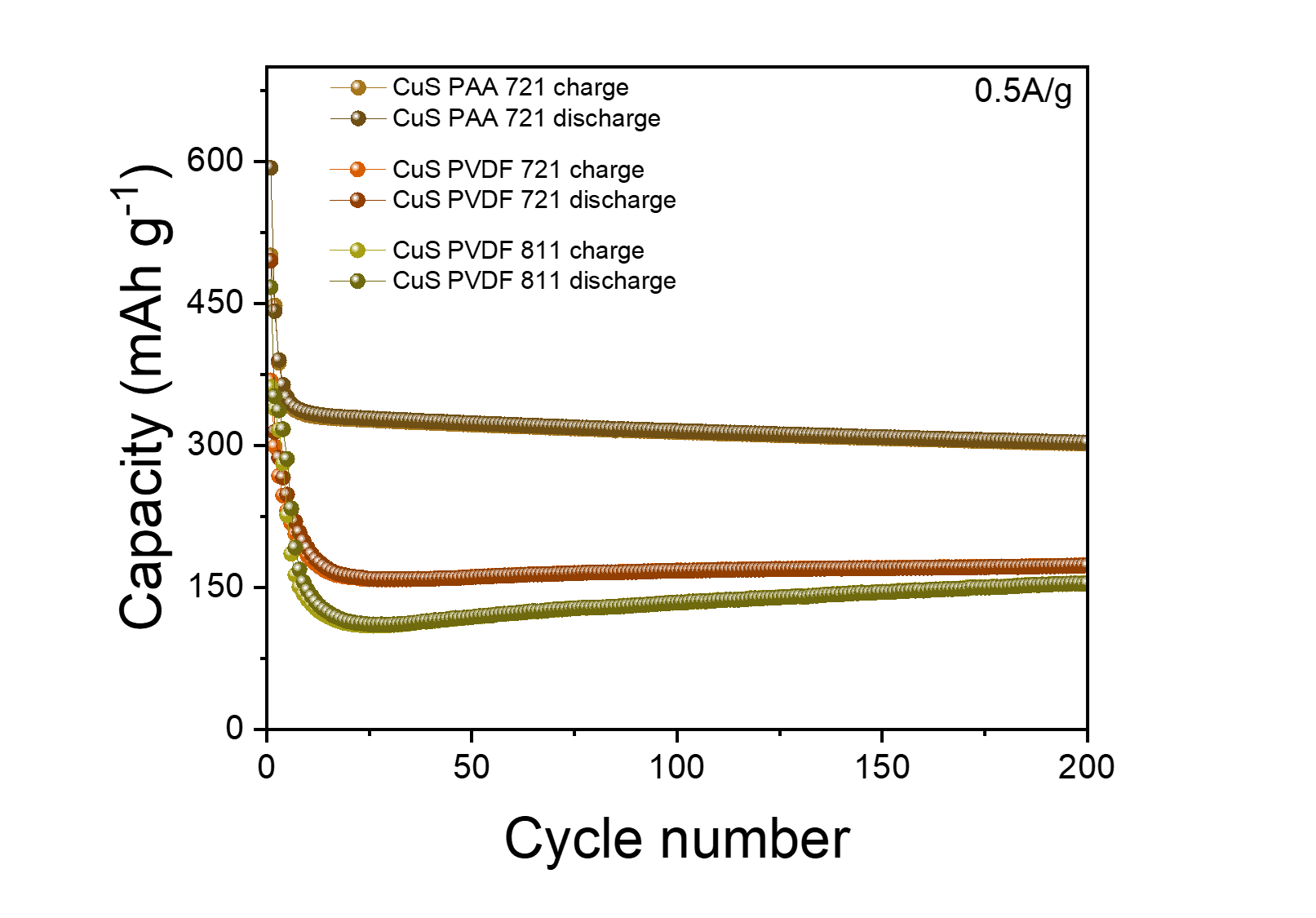
**

**Figure S29.** Comparison of cycling performance depending on binder type and the active material/conductive agent/binder ratio using the c-Al-CC. PAA binders exhibit enhanced cycling performance compared to PVDF binders, and a higher content of conductive additive yields improved cycling performance.


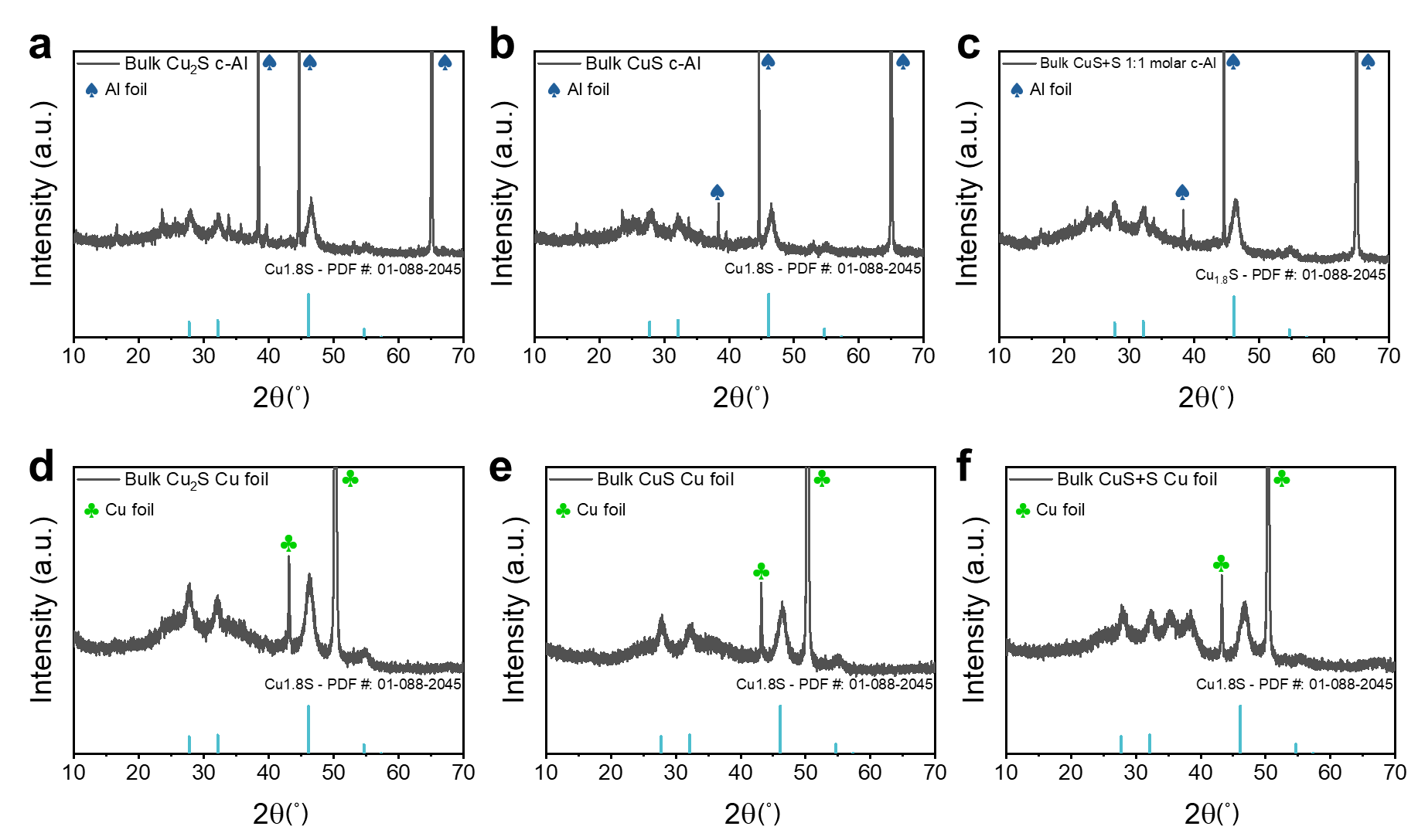


**Figure S30.** Ex**-**situ XRD patterns of **(a)** bulk Cu_2_S, **(b)** bulk CuS, and **(c)** CuS+S electrode supported on a c-Al-CC after 300 cycles. Ex**-**situ XRD patterns of **(d)** bulk Cu_2_S, **(e)** bulk CuS, and **(f)** CuS+S electrode supported on a Cu-CC after 300 cycles.

**
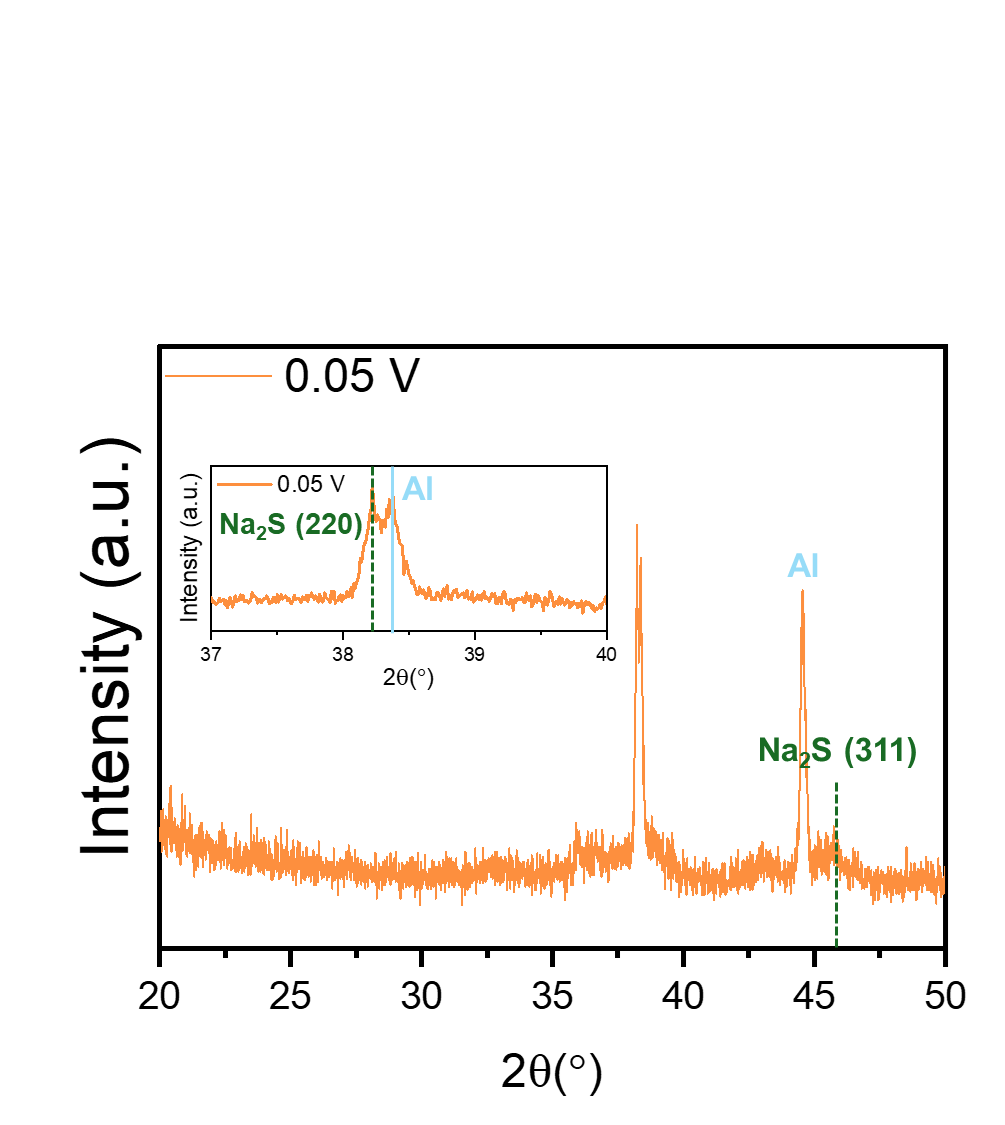
**

**Figure S31.** Ex-situ XRD pattern of bulk CuS electrode with c-Al-CC at 100^th^ fully discharged state (0.05 V).


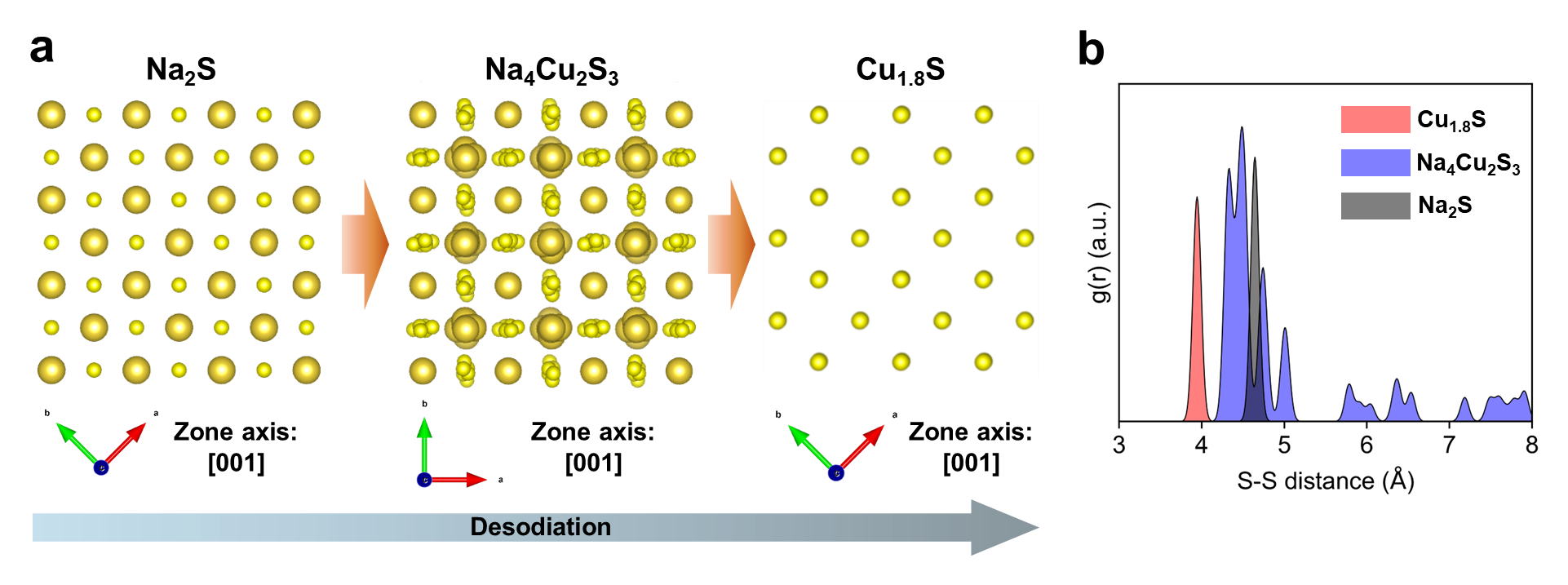


**Figure S32.** (a) Diagram showing sodium (large atom) and sulfur (small atom) among the crystal structures of phase Na_2_S, Na_4_Cu_2_S_3_, and Cu_1.8_S viewed from the c-axis (zone axis: [001]). (b) Radial distribution function (RDF) of S-S bonding distance of Cu_1.8_S, Na_4_Cu_2_S_3_, and Na_2_S. Na_2_S, Na_4_Cu_2_S_3_, and Cu_1.8_S shares a highly similar sulfur atomic framework. Furthermore, the S-S bonding distance in Na_4_Cu_2_S_3_ lies between those of Na_2_S and Cu_1.8_S, suggesting that the phase transition is facilitated by the structural similarity of the crystal lattice during the desodiation process.

**References**

[1] G. Kresse, J. Furthmü, *Efficient iterative schemes for ab initio total-energy calculations using a plane-wave basis set*, 1996.

[2] J. P. Perdew, K. Burke, M. Ernzerhof, *Generalized Gradient Approximation Made Simple*, 1996.

[3] H. J. Monkhorst, J. D. Pack, *Special points for Brillonin-zone integrations**, Vol. 13, 1976.

[4] K. Momma, F. Izumi, *J Appl Crystallogr* 2008, *41*, 653.

[5] H. Li, H. Zhang, M. Zarrabeitia, H. P. Liang, D. Geiger, U. Kaiser, A. Varzi, S. Passerini, *Adv Sustain Syst* 2022, *6*.

[6] M. Ye, X. Wen, N. Zhang, W. Guo, X. Liu, C. Lin, *J Mater Chem A Mater* 2015, *3*, 9595.
